# Predicting the geographical distributions of the macaque hosts and mosquito vectors of *Plasmodium knowlesi* malaria in forested and non-forested areas

**DOI:** 10.1186/s13071-016-1527-0

**Published:** 2016-04-28

**Authors:** Catherine L. Moyes, Freya M. Shearer, Zhi Huang, Antoinette Wiebe, Harry S. Gibson, Vincent Nijman, Jayasilan Mohd-Azlan, Jedediah F. Brodie, Suchinda Malaivijitnond, Matthew Linkie, Hiromitsu Samejima, Timothy G. O’Brien, Colin R. Trainor, Yuzuru Hamada, Anthony J. Giordano, Margaret F. Kinnaird, Iqbal R. F. Elyazar, Marianne E. Sinka, Indra Vythilingam, Michael J. Bangs, David M. Pigott, Daniel J. Weiss, Nick Golding, Simon I. Hay

**Affiliations:** Spatial Ecology & Epidemiology Group, The Big Data Institute, Li Ka Shing Centre for Health Information and Discovery, University of Oxford, Oxford, OX3 7BN UK; Spatial Ecology & Epidemiology Group, Department of Zoology, University of Oxford, Oxford, OX1 3PS UK; Department of Social Sciences, Oxford Brookes University, Oxford, OX1 0BP UK; Department of Zoology, Faculty of Resource Science and Technology, Universiti Malaysia Sarawak, 94300 Kota Samarahan, Sarawak Malaysia; Departments of Zoology and Botany, University of British Columbia, Vancouver, BC V6T 1Z4 Canada; Biodiversity Research Centre, University of British Columbia, Vancouver, BC V6T 1Z4 Canada; Primate Research Unit, Department of Biology, Faculty of Science, Chulalongkorn University, Bangkok, 10330 Thailand; Fauna & Flora International, Singapore, 247672 Singapore; Institute for Global Environmental Strategies, Kamiyamaguchi 2108-11, Hayama-cho, 240-0115 Kanagawa Japan; Wildlife Conservation Society, Mpala Research Center, Nanyuki, 10400 Kenya; Research Institute for the Environment and Livelihoods, Charles Darwin University, Northern Territory, 0909 Australia; Faculty of Science and Technology, Federation University Australia, Mt Helen, Victoria 3350 Australia; Evolutionary Morphology Section, Primate Research Institute, Kyoto University, Inuyama, Japan; Field Conservation Program, S.P.E.C.I.E.S., Ventura, CA USA; Conservation Science Program, Tiger Creek Wildlife Refuge, Tyler, TX USA; Eijkman-Oxford Clinical Research Unit, Jakarta, Indonesia; Department of Parasitology, Faculty of Medicine, University of Malaya, Kuala Lumpur, Malaysia; Department of Entomology, Faculty of Agriculture, Kasetsart University, Bangkok, 10900 Thailand; Public Health and Malaria Control Department, International SOS, Jalan Kertajasa, Kuala Kencana, Papua, 99920 Indonesia; Institute for Health Metrics and Evaluation, University of Washington, Seattle, WA 98121 USA; Wellcome Trust Centre for Human Genetics, University of Oxford, Oxford, OX3 7BN UK

**Keywords:** Species distribution model, Deforestation

## Abstract

**Background:**

*Plasmodium knowlesi* is a zoonotic pathogen, transmitted among macaques and to humans by anopheline mosquitoes. Information on *P. knowlesi* malaria is lacking in most regions so the first step to understand the geographical distribution of disease risk is to define the distributions of the reservoir and vector species.

**Methods:**

We used macaque and mosquito species presence data, background data that captured sampling bias in the presence data, a boosted regression tree model and environmental datasets, including annual data for land classes, to predict the distributions of each vector and host species. We then compared the predicted distribution of each species with cover of each land class.

**Results:**

Fine-scale distribution maps were generated for three macaque host species (*Macaca fascicularis*, *M. nemestrina* and *M. leonina*) and two mosquito vector complexes (the Dirus Complex and the Leucosphyrus Complex). The Leucosphyrus Complex was predicted to occur in areas with disturbed, but not intact, forest cover (> 60 % tree cover) whereas the Dirus Complex was predicted to occur in areas with 10–100 % tree cover as well as vegetation mosaics and cropland. Of the macaque species, *M. nemestrina* was mainly predicted to occur in forested areas whereas *M. fascicularis* was predicted to occur in vegetation mosaics, cropland, wetland and urban areas in addition to forested areas.

**Conclusions:**

The predicted *M. fascicularis* distribution encompassed a wide range of habitats where humans are found. This is of most significance in the northern part of its range where members of the Dirus Complex are the main *P. knowlesi* vectors because these mosquitoes were also predicted to occur in a wider range of habitats. Our results support the hypothesis that conversion of intact forest into disturbed forest (for example plantations or timber concessions), or the creation of vegetation mosaics, will increase the probability that members of the Leucosphyrus Complex occur at these locations, as well as bringing humans into these areas. An explicit analysis of disease risk itself using infection data is required to explore this further. The species distributions generated here can now be included in future analyses of *P. knowlesi* infection risk.

**Electronic supplementary material:**

The online version of this article (doi:10.1186/s13071-016-1527-0) contains supplementary material, which is available to authorized users.

## Background

Approximately one million malaria cases were reported by countries in the Indochinese Peninsula, Malay Peninsula and insular southeast Asia in 2013, and the *Plasmodium knowlesi* parasite was the most common cause of malaria in Malaysia [1]. It is a zoonotic disease that can cause severe symptoms and fatalities in humans [2], and is transmitted among macaques and to humans by anopheline mosquitoes [3]. Outside Malaysia, human cases have been reported from a small number of dispersed locations [3, 4] but the distribution of *P. knowlesi* in these countries is largely unknown. There are far more reports of macaque and mosquito populations, which provides an opportunity to use these distributions to refine estimates of the geographical distribution of knowlesi malaria.

A World Health Organization consultation concluded that this disease is a public health problem that is limited to population groups that live, work in or visit forested areas [5] and it is commonly cited as such [6–10]. No study has, however, analysed the relationship between forest cover and the distributions of the primary *P. knowlesi* host or vector species, across the ranges of these species, limiting our ability to understand the risk factors for disease transmission.

*Plasmodium knowlesi* parasites regularly infect *Macaca fascicularis* and *M. nemestrina* macaques [11, 12] and detailed molecular studies have shown that recent human infections in Malaysia match two distinct populations of parasites found in *M. nemestrina* and *M. fascicularis* respectively [8, 13]. *Macaca leonina* is a close relative of *M. nemestrina* that has only recently been classified as a separate species [14, 15]. The distribution of *M. leonina* extends further north than either *M. fascicularis* or *M. nemestrina* to areas of north Myanmar where *P. knowlesi* cases have been found [16, 17]. For this reason, *M. leonina* has been included with *M. nemestrina* on previous maps of *P. knowlesi* risk that display overlapping ranges of the species involved [3] and is considered a putative host species.

There is good evidence that *P. knowlesi* is transmitted to humans by a number of mosquito species from the Leucosphyrus Group: *Anopheles dirus* [18] and *An. cracens* [19] in the Dirus Complex, and *An. latens* [20], *An. balabacensis* [21] and *An. introlatus* [22] from the Leucosphyrus Complex. Earlier studies did not identify mosquitoes to the species level using molecular methods but they add to the body of evidence that members of the Leucosphyrus Group transmit this malaria from monkeys to humans [23]. Indeed, no mosquito species outside the Leucosphyrus Group has so far been implicated by studies conducted in the field.

Previous approaches that superimposed potential host and vector species range maps [3, 4] do not provide insight into the variation in *P. knowlesi* infection risk within these ranges and do not provide an evidence base for the potential link between forest cover and disease risk. We used species distribution models to investigate the distributions of each of the known and putative hosts and vectors of *P. knowlesi* parasites, and to explore their relationships with forest cover, forest use, and other rarely considered but potentially influential environmental variables. Our aim was to produce predicted species distributions, based on empirical data, that could be used in future studies, combined with what data there is on *P. knowlesi* infections at different locations, to predict geographical variation in disease risk in future studies.

## Methods

### Summary

We used a boosted regression tree (BRT) species distribution model, constructed in R, to examine the relationship between the occurrence of each macaque and mosquito species and 19 environmental covariates, and to predict the relative probability of occurrence for each species at every square (pixel) in a 5 × 5 km grid. The data used by the model were (i) occurrence data points for each species; (ii) survey location datasets that described the sampling bias in the occurrence data; and (iii) a suite of environmental variables. During the study period (1990 to 2014), deforestation has led to dramatic changes in forest cover so we constructed annual data layers for each land cover class. Finally, the model outputs on the islands of the archipelago were masked by a range defined for each species.

### Species occurrence data collation

For each species investigated, we undertook a wide literature search for reports of occurrence and then applied inclusion criteria to ensure the data quality met our minimum standards.

For each macaque species, we conducted a Web of Science bibliography search using the species name (including common names: long tailed macaque, crab eating macaque, kra macaque, pig tailed macaque). We searched the resulting articles for (i) reports of the species found at specific locations and (ii) citations for other sources of occurrence data. We also wrote to the study authors to request unpublished datasets. Finally, we asked conservation organisations working in the region for their unpublished data.

Inclusion criteria for the macaque occurrence data were (i) reports from a specified date on or after 1 January 1990 and ideally on or after 1 January 1999 to match, as closely as possible, the year ranges for the covariate data; (ii) reports from a specific location representing an area not greater than 5 × 5 km; (iii) individual species identified; and (iv) reports of free-living macaques not captive animals. Aggregated data from multiple time periods or multiple sites were disaggregated to single time periods and sites. Duplicate records of the same species found at the same site within the same year were removed, with a single record for that year retained, in order to mitigate against over-sampling at specific sites. An annual time period was chosen to match the land cover data, which were generated for every year.

Coordinates provided by data contributors were converted into decimal degrees. Sites without coordinates from the data provider were assigned coordinates by identifying the site in at least two online gazetteers (GeoNames, Google Earth, Google Maps, iTouchMap and/or OpenStreetMap).

The mosquito data collation mirrored the process for macaque data collation above and has been reported previously [24]. Low volumes of data were available for most species so we also collated data for the relevant species complexes (the Leucosphyrus Complex and the Dirus Complex). The previous mosquito data collection was extended to include reports published up to November 2015.

In total, we collated 1,116 occurrence records between 1999 and 2014 for *M. fascicularis* and 1,025 for *M. nemestrina*. The inclusion criteria that locations should not be greater than 5 × 5 km was relaxed for *M. leonina* and we collated 450 records from 1992 to 2014, of which 33 were linked to locations representing areas > 25 km^2^. The borders of each area > 25 km^2^ were defined in ArcMap.

We collated 545 records for the Dirus Complex (including 107 for *An. dirus* and 19 for *An. cracens*) and 49 for the Leucosphyrus Complex (including 21 for *An. balabacensis*, 11 for *An. latens*, and 9 for *An. introlatus*) from 1982 to 2013.

Collectively the surveys used did not sample a representative set of environments at the same frequency that each environment occurs within the study area, for example camera traps used by conservationists are rarely set up in urban areas or impenetrable jungle. Our aim when collating the background datasets was to account for as much of the sampling bias in the presence data as possible by selecting datasets that used the same methods (e.g. camera traps and direct observations from transect walks) to record similar species (primates and other mammals). The surveys that provided presence data for the macaque and anopheline species frequently reported more than one species and met our criteria for background data of using the same methods to record similar species.

We obtained the locations of all mammal surveys held by the Global Biodiversity Information Facility (www.gbif.org) that were conducted on a specified date from 1990 onwards, at a specified location and within our area of study. We also used the records from the other two macaque species to generate additional background data for the species being modelled. Each background dataset was restricted to the range (plus a 300 km buffer to allow for uncertainty in the ranges) of the species being modelled.

To account for the sampling bias in the anopheline datasets, we used a database of all published malaria vector surveys held by the Malaria Atlas Project (www.map.ox.ac.uk/explorer). Each background dataset was restricted to the range (plus a 300 km buffer to allow for uncertainty in the ranges) of the species, complex or group being modelled.

Table 1 provides the total number of presence and background data points available for each macaque and mosquito species and complex. The full distributions of these datasets, in space and time, are shown in Additional file 1.

**Table 1:** Number of data points used in each model

	No. presence points	No. background points
*M. fascicularis*	1, 116	2, 267
*M. nemestrina*	1, 025	608
*M. leonina*	450	1, 041
*An dirus*	107	447
Dirus Complex	545	881
Leuco. Complex	49	913
Leuco. Group	615	1, 802

### Covariate data surface construction

Nineteen environmental and human population variables were tested in the species distribution models and are summarised in Additional file 2. The process of constructing environmental data layers from MODIS satellite data has been described previously [25] and was extended from Africa to the rest of the world, covering the period 2000 to 2014. In addition, to include seasonality in temperature and moisture/vegetation indices, the standard deviation of the monthly values at each location was calculated. Of the 19 land cover classes, six that were not relevant to the study area were excluded. Forest data layers were constructed separately (see below) and the urban class was excluded because we used human population density data, leaving a total of seven classes (open shrubland, woody savannah, savannah, grassland, permanent wetlands, cropland, and cropland-natural vegetation mosaic).

### Annual intact and disturbed forest data surface construction

Total forest cover was defined annually from 2001 to 2012 by combining the five forest classes available in the International Geosphere and Biosphere Programme (IGBP) land cover dataset produced using MODIS satellite imagery (MCD12Q1) [26]. We divided the aggregated forest cover data into two sub-classes defined previously by forest landscape researchers [27]; intact forest and disturbed forest. The Intact Forest Landscape (IFL) map for the year 2000 is a publicly available vector dataset encompassing areas defined as ‘an unbroken expanse of forest showing no signs of human activity and having an area of at least 500 km^2^’ [27], which we converted to a 500 m spatial resolution raster dataset. The IGBP forest cover data for 2001 was divided between the two sub-classes using the IFL dataset. The IGBP forest cover data for subsequent years was then used to reclassify any cells in the preceding year that had been considered forest (intact or disturbed) to non-forest, if the corresponding IGBP cell showed a transition from forested to non-forested land cover from one year to the next. If the IGBP data showed a transition from non-forest to forest, the cell was defined as disturbed forest in our data layers based on the assumption that new forest regrowth would not meet the criteria for intact forest within this time period. The annual products were produced sequentially, with results from the preceding year used to create those for the subsequent year, thus producing output that tracked the decline in forest cover and any areas of regrowth. Once produced, the 500 m categorical results for each year were converted to fractional (i.e. continuous) products at a 5 km resolution, with values ranging from 0.0 (no forest cover) to 1.0 (complete forest cover) for the proportion of each 5 × 5 km classified as each forest type. Further details on the construction and validation of the forest covariate data surfaces are provided in Additional file 2.

### Model

The boosted regression trees method used here is a variant of the model used in previous analyses of malaria vector species [24] and diseases such as dengue [28]. Boosted regression tree modelling combines both regression trees (which build a set of decision rules on the predictor variables by portioning the data into successively smaller groups with binary splits) and boosting (which selects sets of trees that minimise the loss of function) to best capture the variables that define the distribution of the input data [29–31]. The core setup used in the current study has been described previously [28]. The changes made to the method for this study allowed temporal changes in land cover to be incorporated and improved the way absence data and sampling bias were handled. Our methods for handling polygon data also varied from those used previously.

The previous approaches [24, 28] used synoptic covariate values that covered a period of several years. In this study, we incorporated temporal covariate data for the land cover classes so that the year of occurrence was taken into account. We were able to construct covariate data layers for each year from 2001 to 2012 but the species data available for this period did not cover all of the geographical regions for which we had data. To test the impact of using species data that could not be matched to the corresponding annual land cover layers we constructed two test datasets for *M. leonina* and the Dirus Complex for the time period 2001 to 2012 only. We ran the model as described below twice on each dataset; once linking the species data to annual covariate layers and once linking the species data to the 2012 covariate layers only. The results provided in Additional file 3 show that the outputs from the two model versions were similar but the version using annual covariates performed slightly better. We therefore used the full dataset for the final model runs, including data outside the 2001 to 2012 year range, in order to maximise the spread of species data used, and we linked species data from 2001 to 2012 to covariate values for the relevant year in order to improve the predictions where possible. For all occurrences prior to 2001, covariate values for 2001 were used, and for any data collected after 2012, covariate values for 2012 were used. Model predictions were made to the most contemporary covariate data available.

The boosted regression trees method requires both presence and absence data. Pseudo-absence data, also known as background data, are generated when true absence data is not available. The vast majority of species occurrence datasets are subject to spatial bias, for example, areas near roads and paths may be more likely to be surveyed than other sites. If unaccounted for, this survey bias can translate into environmental bias in the fitted model. One approach for coping with biased occurrence data is to select background data that reflect the same survey bias as the occurrence data. The resulting model should identify suitable environments for the species within the sampled space, rather than just areas that are more heavily sampled. This approach does not eliminate sampling bias issues entirely but improved model performance has been demonstrated when compared to the use of randomly selected background data [32]. For the work presented here, mammal and malaria vector occurrence records from within the study area were used as pseudo-absence data for the macaque and vector models, respectively. These background datasets were chosen because the sampling methods were the same as those used for the target species.

For each species, we fitted 120 submodels each trained to a randomly selected bootstrap of the presence/background dataset. Each bootstrap contained a minimum of five presence and five background points. To account for uncertainty in the geographic location of occurrences linked to polygon locations > 25 km^2^ in the *M. leonina* dataset, one 5 × 5 km pixel within each polygon was randomly selected for each bootstrap. Each of the submodels generated a predicted value for the relative probability of species occurrence at every 5 × 5 km pixel and together the submodels generated a distribution of predicted values for every pixel. We generated maps displaying the mean, 0.025 quantile and 0.975 quantile values from these distributions for each pixel.

To evaluate the ensemble’s predictive performance, we calculated, for each submodel, the area under the receiver operator curve (AUC), i.e. the area under a plot of the true positive rate versus the false positive rate, reflecting the ability to discriminate between presence and background records, whilst marginalising the arbitrary choice of a classification threshold [33]. For each submodel, we reported the mean AUC under fivefold cross-validation using a pairwise distance sampling procedure to remove spatial sorting bias in the model validation [34]. We then combined these submodel validation statistics to obtain an overall estimate of predictive performance in the ensemble, and uncertainty in this estimate.

There were insufficient presence data for *An. cracens*, *An. balabacensis*, *An. latens*, or *An. introlatus* to model these species individually so members of the Dirus and Leucosphyrus Complexes were modelled collectively to predict the relative probability of one or more of the species within a complex occurring.

### Covariate density plots

To illustrate the relationships between the coverage of each land cover class and predicted species occurrence, we plotted the relative density of pixels at each percentage coverage for all pixels where the relative probability of species occurrence was greater than 0.75. This threshold was selected in order to identify pixels with a high probability of occurrence rather than simply those with a relative probability greater than 50 %. The density values were calculated from the ratio of the number of pixels where the relative probability of species occurrence was greater than 0.75 to the total number of pixels in the study area.

### Masks

The model outputs for each species were restricted to the islands within its known range, using the range maps developed for this project. For the macaque species, range maps were obtained from the International Union for the Conservation of Nature [35]. These ranges did not incorporate all new data or introduced populations. We therefore used our occurrence dataset to adjust the range for each species by either dragging the range out to encompass new reports next to the existing range or by identifying the borders of any confirmed population that was not contiguous with the existing range. For this exercise, data did not need to meet the criteria of representing an area < 25 km^2^. For the mosquito species/complexes, we used range maps previously published by three groups [24, 36, 37] and updated these in the same way as the macaque ranges. In places where the three ranges differed for a particular species, we selected the broadest of the available options.

## Results

### Macaque and mosquito distributions

**Fig. 1:**
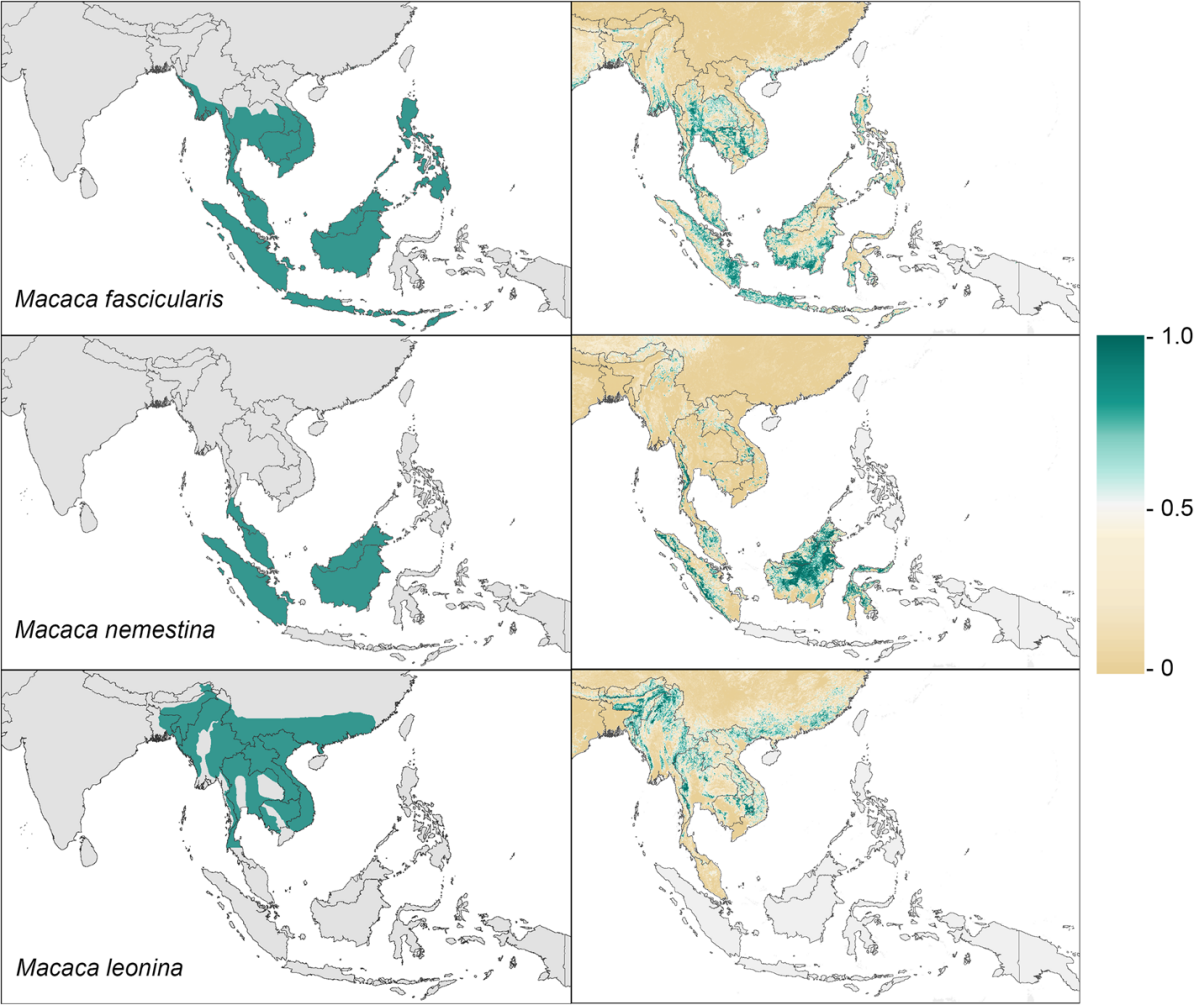
Ranges and predicted distributions of the macaque species. The three maps on the *left* show the current range of each macaque species and the three maps on the *right* show the predicted relative probability of occurrence at every 5 × 5 km pixel within the study area on a scale of 0 to 1.0

**Fig. 2:**
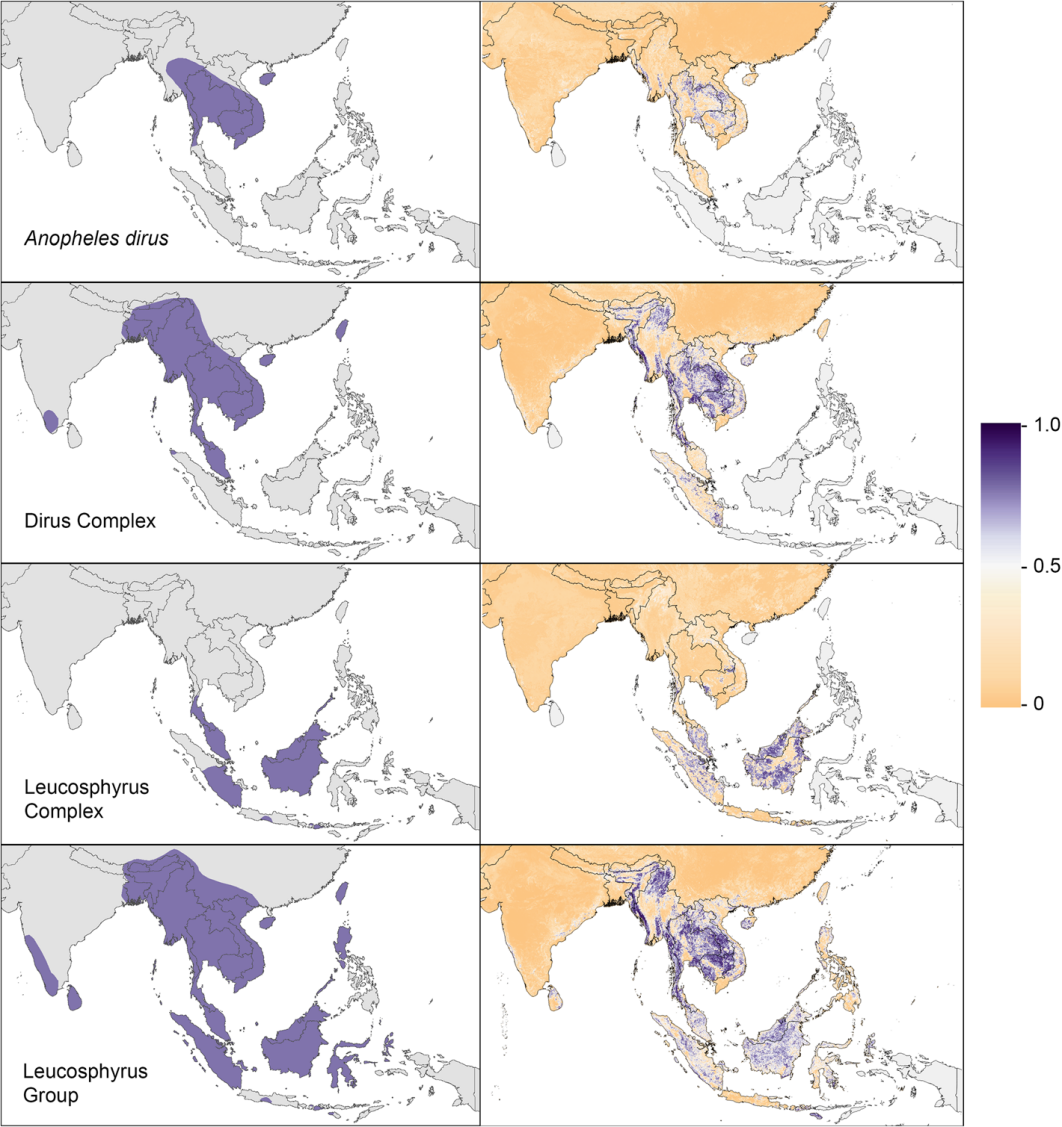
Ranges and predicted distributions of the mosquito species, complexes and group. The four maps on the *left* show the current range of each mosquito species, complex or group, and the four maps on the *right* show the predicted relative probability of occurrence at every 5 × 5 km pixel within the study area on a scale of 0 to 1.0

The mean model outputs, masked out on islands outside the species range, were used to generate predicted species distribution maps (Figs. 1 and 2). The AUC values ± standard error for the predicted macaques distributions were 0.858 ± 0.001 for the *M. fascicularis* map, 0.821 ± 0.003 for the *M. nemestrina* map and 0.830 ± 0.002 for the *M. leonina* map. The AUC values ± standard error for the predicted mosquito distributions were 0.860 ± 0.005 for the *An. dirus* map, 0.885 ± 0.002 for the Dirus Complex map, 0.842 ± 0.009 for the Leucophyrus Complex map and 0.883 ± 0.001 for the Leucophyrus Group map. The Leucosphyrus Complex predictions should be interpreted with caution because the data volume for this Complex was low and the data was sparse. The model did not predict many areas with a high probability of occurrence outside the current macaque ranges, indicating that each species has largely realised its predicted niche, excluding islands that have not yet been populated (Fig. 1).

The 0.025 and 0.975 quantile from the model ensemble, and the top predictors for each species together with values for their relative influence on the model, are provided in Additional files 4 and 5.

Density plots illustrating the ratio of proportional land cover in areas with high predicted probability of occurrence (predictions of 0.75 and above) to proportional land cover in all areas are given in Additional file 6. If all land cover proportions (for example high, low and no coverage of grassland) were equally likely to be suitable (a null distribution), each of these density plots would be flat. High or low regions of the density plots therefore indicate proportional land cover values which are predicted to be more or less suitable for occurrence of the species in question. These plots should not be interpreted as providing robust evidence for any specific relationship between each land cover class and species presence, in part because each plot is influenced by the relationships between species occurrence and all of the other covariates that went into the model for the locations where we had data. The purpose of these plots is solely to provide an extra visualisation of the predicted species distribution results and supplement the information that can be visualised directly on the maps in Figs. 1 and 2.

### Data availability

All data used and generated by the project are publicly available. The vector occurrence data used in our models are available from http://www.map.ox.ac.uk/explorer/#EntityPlace:Anopheline. The macaque occurrence datasets used in each model are provided in Additional files 7, 8 and 9. The GeoTIFF files containing mean model outputs as displayed in the distribution maps are provided in Additional files 10, 11, 12, 13, 14, 15 and 16. The model code generated is available on an open source basis from https://github.com/SEEG-Oxford/seegSDM.

## Discussion

Forest cover is shrinking in southeast Asia and the loss of natural or intact forest is in part due to conversion to degraded or logged areas and plantations (included in the disturbed forest category in this study) [38]. Furthermore, this trend is expected to continue [39]. This study is the first to predict the full distributions of knowlesi malaria host and vector species by modelling these species across their ranges using environmental data surfaces that track changes in land cover. The predicted distributions generated are not solely restricted to forested areas and any disease risk in non-forested areas has the potential to dramatically increase estimates of the population at potential risk of knowlesi malaria within this densely populated region [5].

The species distributions were generated using environmental covariates but the relationship between species occurrence and these covariates may vary in time and space. One advantage of the BRT approach is its ability to fit a single overall model to multiple distinct patterns within the data. This flexible nonparametric statistical model is better able to simultaneously model multiple environmental relationships than more traditional approaches [29]. The cost of this flexibility and focus on predictive power is that it complicates inference, thus, it is not possible to identify causal relationships between the environmental covariates and the species modelled. For the vector species, data scarcity meant we had to pool data from multiple species and again BRT’s ability to encompass different relationships within a pooled dataset helps to overcome some of the issues of modelling multiple species together. The model is still limited as it is only able to model relationships for the combinations of covariates found within the field data provided to the model.

The model is also limited by the set of covariates used. These do not capture all potential sources of variation that may influence the distributions of these species in some or all parts of their ranges. For example, forest edge effects were not incorporated in our study. Previously the booted macaque, *M. ochreata*, has been shown to be more abundant near forest edges at two sites in Sulawesi [40], however, a study of tiger prey, including *M. nemestrina*, on Sumatra found that edge effects associated with national park boundaries were not significant once human population density was considered [41].

The potential predictors provided to the model are also limited by the fact that each land class encompasses variation in that habitat; most noticeably the disturbed forest class includes natural forest with evidence of human disturbance, or less than 500 km^2^ in area, and established palm and rubber plantations. Our aim was to model each species across its entire range using region-wide covariate datasets rather than more detailed but locally-restricted data. Furthermore, our approach did not incorporate factors that may be important at finer resolutions than the 5 km used here [42]. Nevertheless, the models performed reasonably well using the covariate data that we constructed for the region as a whole, at a 5 km resolution, and gave AUC values of 0.82 and above. The answers to more detailed questions about the influence of individual factors on host and vector populations require more detailed studies. The distributions presented here provide a good estimate of the full distributions of the species important to *P. knowlesi* transmission across their ranges.

Earlier studies of *M. nemestrina* were more detailed but also more geographically restricted. In Indonesian Borneo, *M. nemestrina* was found in intact and partially degraded or burned forest but was absent from completely deforested areas [43]. On Sumatra, *M. nemestrina* densities were higher in areas of low human population density [41] and this species was less common in plantations compared to *M. fascicularis,* although it was found [44]. *Macaca nemestrina* raids rice crops in Sumatra, most frequently on farms close to the forest edge [45]. Of the macaque species studied here, our model predicted a high relative probability of *M. nemestrina* presence in the smallest number of land cover classes, and rarely predicted occurrence in non-forested areas. This finding and those of other studies support the hypothesis that this species will be negatively impacted by conversion of forested areas to non-forest habitats but conversion of intact forest to disturbed forest will allow populations to remain. The latter conversion will, however, bring humans into the vicinity of these macaque populations where they had previously been separated.

Our model predicted that the *M. fascicularis* and *M. leonina* distributions encompass a wider range of habitats than the *M. nemestrina* distribution, and this is particularly apparent for the *M. fascicularis* distribution. A number of studies have measured the habitat preferences of *M. fascicularis* within restricted parts of its range but none have considered the full distribution of this species. In Malaysian Borneo, studies found that *M. fascicularis* populations were initially negatively impacted by logging activities but their local abundance was higher in areas that had been logged ten years previously than in unlogged forest [46]. In Vietnam, *M. fascicularis* was found in public parks and temples as well as mangroves, river banks and primary, disturbed and secondary forests [47]. On Sumatra, *M. fascicularis* was found in plantations more commonly than *M. nemestrina* [44]. In Thailand, *M. fascicularis* habitats have changed from natural forests to temples and parks over the last 30 years [48] and groups are found in suburban Bangkok, the Thai capital [49]. This species will also freely enter suburban areas of Selangor State, Malaysia [50]. In Singapore, *M. fascicularis* is found at forest perimeters and in forest fragments, where these macaques are habituated to human presence and will leave forest areas for urban habitats [51]. Our results and these previous findings strongly suggest that *M. fascicularis* populations are able to occupy a wide range of habitats. Importantly, the distribution of this species encompasses many locations close to human habitation (urban areas) or activity (disturbed forests, orchards, croplands, etc).

There are fewer studies of *M. leonina* habitat preferences. In recent surveys, *M. leonina* monkeys were found in a range of habitats in Laos from river areas to intermediate plains to dry hilly forest [52]. When the habitat preference of this species was measured within a Laos reserve, it was associated with proximity to village areas (average 6 km) as well as with evergreen and deciduous forest cover, lower elevations and higher temperatures [53]. *Macaca leonina* has also been found to move between primary and secondary forest in a Thai reserve [54]. The evidence available, and our own results, suggests that this species occurs in deforested areas where human activity occurs although, like *M. nemestrina*, this species was not associated with urban areas.

The question of whether these latter two macaque species occur in northern Myanmar is of particular relevance because human cases of *P. knowlesi* malaria have been found in people living in Shan State near the border with Yunnan Province, China [16, 17]. If the current ranges used here are accurate then, of the species we mapped for this study, only *M. leonina* is present in northeast Myanmar. Records of macaques in Myanmar are, however, incomplete and may be out of date [55] although a recent survey close to the Yunnan border in Kachin State (to the north of Shan State) found *M. leonina* monkeys but not *M. fascicularis* [56]. The *M. fascicularis* range extends at least as far north as central Myanmar but our results predict that the habitats further northeast are unsuitable for this species (Fig. 1). Further surveys of this region are necessary to confirm the full list of species present and whether they are infected with *P. knowlesi*.

Sulawesi is not in the natural range of the three macaque species studied here although related species are found on the island [57]. *Macaca fasciculari*s and *M. nemestrina* monkeys are kept as pets on Sulawesi [58] and there is an unsubstantiated report of *P. knowlesi* infecting a macaque here [59]. For these reasons we have shown the model predictions for these species on Sulawesi but this area of the map should be interpreted as showing suitable environments for these species should they establish feral populations.

The Leucosphyrus Complex of mosquitoes was predicted to occur in areas with high coverage of disturbed forest but lacking intact forest cover, although the sparse data and low data volumes for this Complex mean these predictions need to be interpreted with caution. This Complex is responsible for *P. knowlesi* transmission in the region where knowlesi malaria is believed to be most common, Malaysian Borneo, and where deforestation (the loss of intact forest) is occurring [21, 60]. Notifications of knowlesi malaria cases increased in Malaysian Borneo between 1992 and 2011 [61] and our results indicate the conversion of intact forest to disturbed forest, and the resulting impact on the probability of encountering members of the Leucosphyrus Complex, could be a factor here. One study in the northern part of Sabah in Malaysian Borneo recently found an association between two forest variables, forest loss and total cover within 2 km of a village, and the estimated incidence of knowlesi malaria at the village level in the two districts studied but they did not distinguish intact and disturbed forest [62]. The small number of previous studies of the Leucosphyrus Complex all focussed on measuring the characteristics of relevance to vectorial capacity rather than relationships with environmental factors. These studies were conducted in restricted geographical areas and did not explicitly measure environmental variables, although sites were classified into types. *Anopheles balabacensis* in an area of Sabah was more abundant in a village site than the forest site and farming site surveyed, but *P. knowlesi* infection rates were lowest in the village [21]. *Anopheles latens* human biting rates in Sarawak, Malaysia were higher at a fruit tree farm on the forest fringe and a forest site compared to a longhouse site, and *P. knowlesi* sporozoite infected individuals were only found at the fruit tree and forest sites [60]. Ours is the first study to investigate the distribution of the Leucosphyrus Complex, generally considered to be a forest species [24, 36, 37] and to have predicted occurrence in areas with disturbed forest cover.

Two previous studies have considered the relationships between the Dirus Complex and environmental factors. The research presented here builds on an earlier project that predicted distributions for all primary human malaria vectors in the region using an older version of the field dataset used here, and similar methods [24]. In the current study we developed the modelling techniques further to handle sampling bias, we extended the range of covariates tested and we incorporated data on temporal changes to land cover since 2001. We found similar relationships with environmental factors to the earlier work and observed a closer match to the known range of the Dirus Complex. An independent study used a different approach to model both the potential niche and the current distribution or realised niche of this Complex [63]. They used temperature and rainfall to define the fundamental niche, and land cover (specifically forest cover in 2005) to model the realised niche or current distribution. Our results did not predict Dirus Complex occurrence exclusively in forested areas but the outputs from the two studies, our predicted distribution and their realised niche map, show similar results. More detailed but geographically restricted studies have considered differences in abundance in different habitat types at a small number of sites but have not explicitly analysed the relationships with environmental variables. The *An. dirus* biting and infection rates did not differ significantly between a forest site and a forest fringe site in Khanh Hoa Province, Vietnam [64], whereas *An. cracens* was more abundant and had a higher human biting rate at an orchard site compared to a forest edge and village site in Pahang State, Malaysia [19]. Our study took data from a much larger number of sites and used quantified environmental variables, but is restricted to species occurrence.

For all potential vector species, and particularly the members of the Leucosphyrus Complex implicated as *P. knowlesi* vectors, more data is needed to define their distributions with confidence. Large-scale systematic sampling of a range of habitats across the region is urgently needed to address this important gap in our understanding of *P. knowlesi* infection risk.

## Conclusions

Together our results for the macaque hosts and the mosquito vectors of *P. knowlesi* malaria suggest that the relative probability of host macaque species and members of the Leucosphyrus Complex occurring in disturbed forest areas, for example, plantations or timber concessions, and vegetation mosaics, will mean these species can co-exist close to human activity. This finding is of most significance in the southern part of our study area (Malaysia, Indonesia, Singapore, Brunei and part of the Philippines) where members of this Complex are the main *P. knowlesi* vectors. The predicted distribution of the long-tailed macaque, *M. fascicularis*, encompassed many more types of human-occupied habitat. This is of most significance in the northern part of its range and our study area (Myanmar, Thailand, Laos, Cambodia and Vietnam) because members of the Dirus Complex are the main *P. knowlesi* vectors here and our model predicts occurrence of these species in areas of open canopy cover (savannah), vegetation mosaics and cropland as well as closed canopy forest.

Characterising the distribution of all component species is an important first step in understanding the distribution of a vector-borne, zoonotic disease when human infection data is lacking. The maps generated here will help identify areas where there may be a *P. knowlesi* disease risk but further information is needed to extrapolate directly from these maps to an index of risk [42]. The next stage of this work, therefore, needs to consider the relationship between *P. knowlesi* infections and a range of risk factors including the fine scale species distributions presented here as well as geographical, environmental and socioeconomic factors. Using a similar modelling framework, the *P. knowlesi* reservoir and vector maps can be used as explanatory variables to test their ability to predict spatial variation in risk of human *P. knowlesi* infections in areas where human disease data is available. The resulting model could then be used to predict human disease risk in areas where both reservoirs and competent vectors are likely to be present but human disease data is scarce or absent. Only then can we consider estimating the population at risk across the region.

## Additional files

Additional file 1: Distributions of the model input data in space and time. The spatial distributions of the species occurrence data and background data are shown on a series of maps and their temporal distributions are shown by a series of histograms. (DOCX 1584 kb) Additional file 2: Environmental variables used in the species distribution models and construction of the forest cover layers. Details of the covariate data used are provided together with their source details. The construction and validation of the intact and disturbed forest layers is described in detail. (DOCX 27 kb) Additional file 3: Investigating the impact of using annual land cover data. The model was run using identical datasets and either 1) year-matched land cover data or 2) 2012 land cover data. The resulting distributions are shown with the AUC ± standard error, and the top predictors with their relative influences. A map showing the difference between the two resulting distributions is also provided. (DOCX 2977 kb) Additional file 4: The 0.025 and 0.975 quantile model predictions, and the top predictors, for each macaque species. For each macaque species the 0.025 and 0.975 quantile model outputs, masked out on islands outside each species range, are provided with the mean AUC (± standard error) and the relative influence of the top predictors for that species. (DOCX 1183 kb) Additional file 5: The 0.025 and 0.975 quantile model predictions, and the top predictors, for each mosquito model. For each mosquito species, complex or group, the 0.025 and 0.975 quantile model outputs, masked out on islands outside each species or complex range, are provided with the mean AUC (± standard error) and the relative influence of the top predictors for that model. (DOCX 1331 kb) Additional file 6: Proportional land cover in areas with high predicted probability of species occurrence. Plots showing the relative density of pixels at each percentage land class coverage for all pixels where the probability of species occurrence was greater than 0.75, for each species. (DOCX 795 kb) Additional file 7:
*Macaca fascicularis* data. Each record of *M. fascicularis* occurrence is provided with a location and date. Duplicate records within a calendar year have been removed. Locations are classed as points (defined as <25 km^2^) or polygons (defined as >25 km^2^). (XLSX 322 kb) Additional file 8:
*Macaca nemestrina* data. Each record of *M. nemestrina* occurrence is provided with a location and date. Duplicate records within a calendar year have been removed. Locations are classed as points (defined as <25 km^2^) or polygons (defined as >25 km^2^). (XLSX 130 kb) Additional file 9:
*Macaca leonina* data. Each record of *M. leonina* occurrence is provided with a location and date. Duplicate records within a calendar year have been removed. Locations are classed as points (defined as <25 km^2^) or polygons (defined as >25 km^2^). (XLSX 63 kb) Additional file 10: Relative probability of *Macaca fascicularis* occurrence. A GeoTIFF raster data layer containing a predicted value (the mean model output) for every 5×5km pixel within SE Asia excluding islands outside the species range. This file can be opened in GIS software (e.g. QGIS, ArcMap, etc) or using the ‘raster’ R package. (TIF 4459 kb) Additional file 11: Relative probability of *Macaca nemestrina* occurrence. A GeoTIFF raster data layer containing a predicted value (the mean model output) for every 5×5km pixel within SE Asia excluding islands outside the species range. This file can be opened in GIS software (e.g. QGIS, ArcMap, etc) or using the ‘raster’ R package. (TIF 4090 kb) Additional file 12: Relative probability of *Macaca leonina* occurrence. A GeoTIFF raster data layer containing a predicted value (the mean model output) for every 5×5km pixel within SE Asia excluding islands outside the species range. This file can be opened in GIS software (e.g. QGIS, ArcMap, etc) or using the ‘raster’ R package. (TIF 3459 kb) Additional file 13: Relative probability of *Anopheles dirus* occurrence. A GeoTIFF raster data layer containing a predicted value (the mean model output) for every 5×5km pixel within SE Asia excluding islands outside the species range. This file can be opened in GIS software (e.g. QGIS, ArcMap, etc) or using the ‘raster’ R package. (TIF 3461 kb) Additional file 14: Relative probability of a member of the Dirus Complex occurring. A GeoTIFF raster data layer containing a predicted value (the mean model output) for every 5×5km pixel within SE Asia excluding islands outside the complex range. This file can be opened in GIS software (e.g. QGIS, ArcMap, etc) or using the ‘raster’ R package. (TIF 3770 kb) Additional file 15: Relative probability of a member of the Leucosphyrus Complex occurring. A GeoTIFF raster data layer containing a predicted value (the mean model output) for every 5×5km pixel within SE Asia excluding islands outside the complex range. This file can be opened in GIS software (e.g. QGIS, ArcMap, etc) or using the ‘raster’ R package. (TIF 4006 kb) Additional file 16: Relative probability of a member of the Leucosphyrus Group occurring. A GeoTIFF raster data layer containing a predicted value (the mean model output) for every 5×5km pixel within SE Asia excluding islands outside the group range. This file can be opened in GIS software (e.g. QGIS, ArcMap, etc) or using the ‘raster’ R package. (TIF 4524 kb)

## Abbreviations

AUC: area under the receiver operator curve
BRT: boosted regression tree
IFL: intact forest landscape
IGBP: International Geosphere and Biosphere Programme
