# Supplementary material for: Predicting the geographical distributions of the macaque hosts and mosquito vectors of Plasmodium knowlesi malaria in forested and non-forested areas
Source: Parasit Vectors. 2016 Apr 28;9:242. doi: 10.1186/s13071-016-1527-0 (PMC4850754; doi:10.1186/s13071-016-1527-0)
Supplement: Additional file 1: — Distributions of the model input data in space and time. The spatial distributions of the species occurrence data and background data are shown on a series of maps and their temporal distributions are shown by a series of histograms. (DOCX 1584 kb) [file 13071_2016_1527_MOESM1_ESM.docx]

**Distributions of the model input data in space and time**

Species presence data is shown in colour on each of the following maps and charts, and background data points used to represent sampling bias in the presence data are shown in white. The maps shows the spatial distribution of the data and the histograms show the count by year.

***Macaca fascicularis* data**


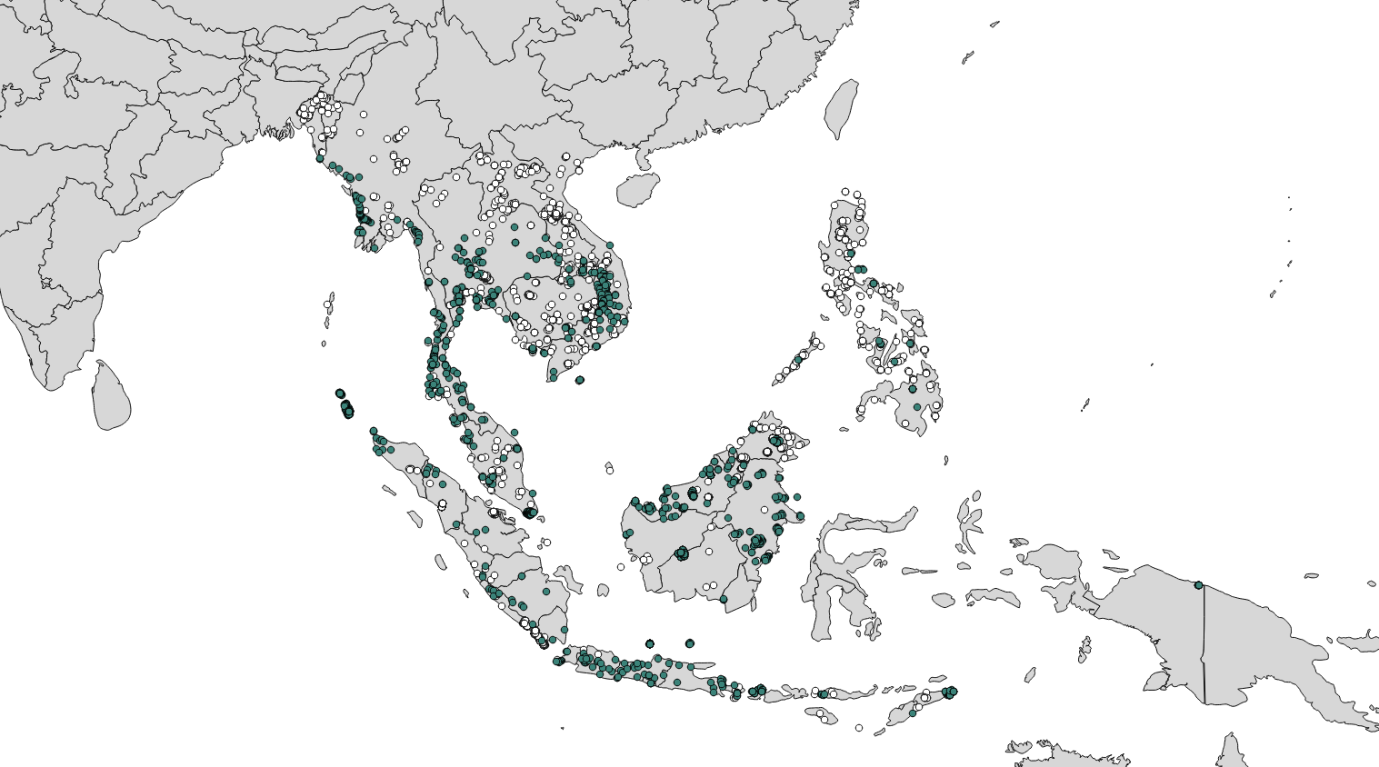


***Macaca nemestrina* data**


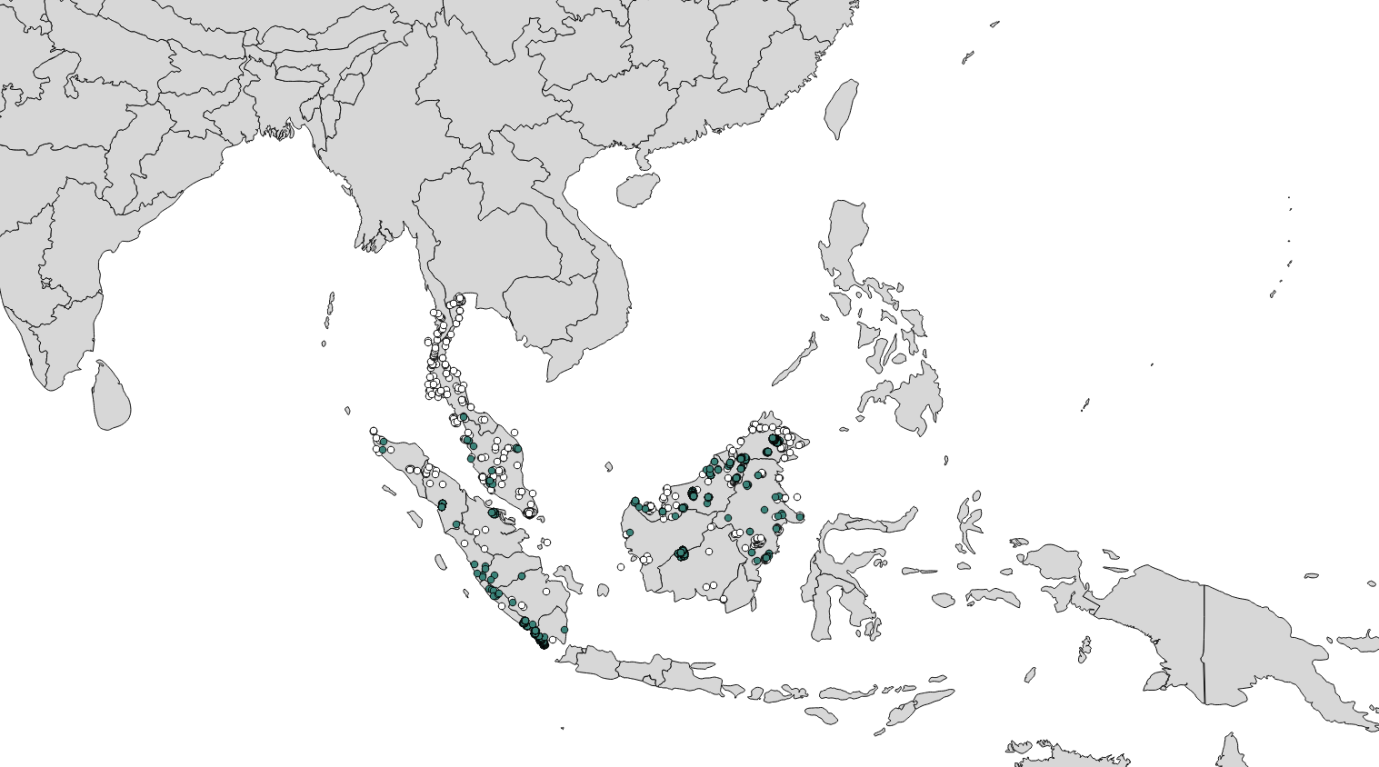


***Macaca leonina* data**


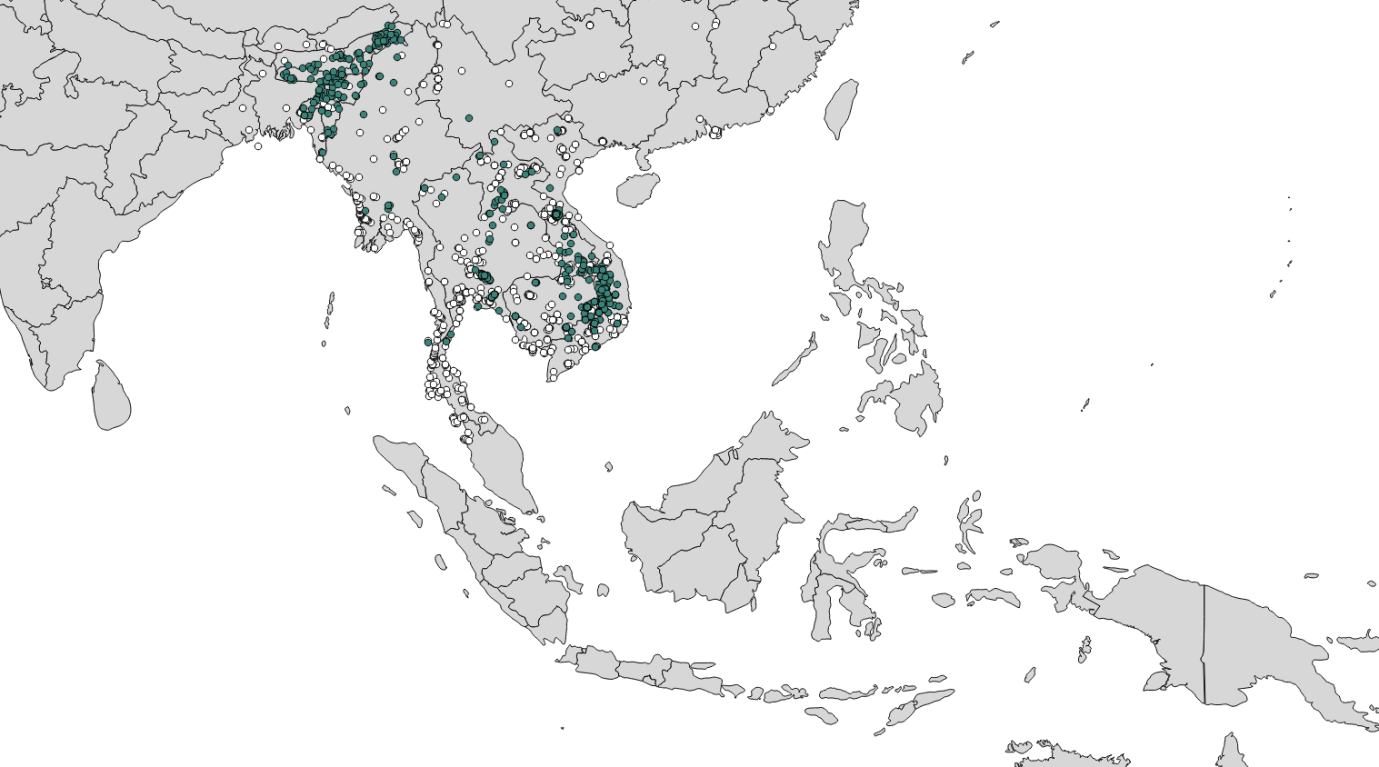


***Anopheles dirus* data**


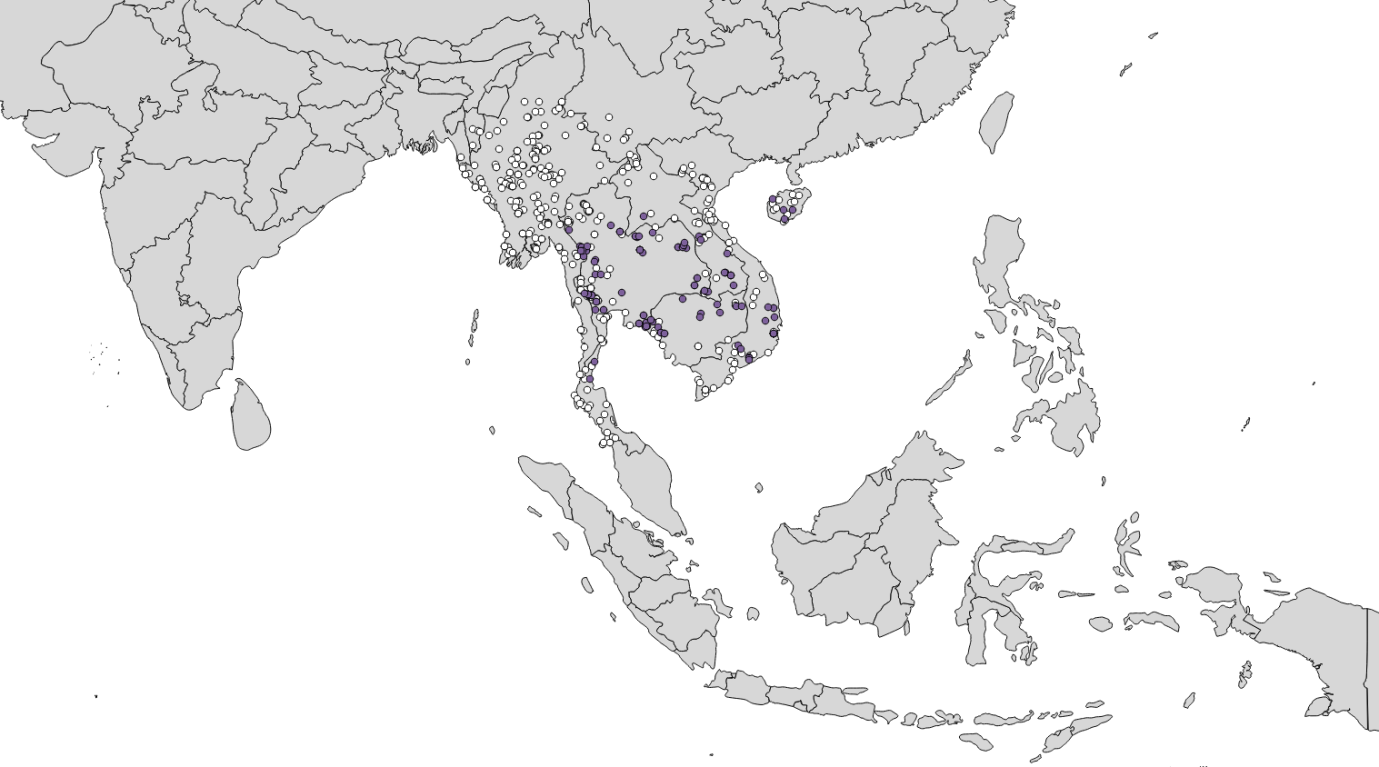


**Dirus Complex data**

**
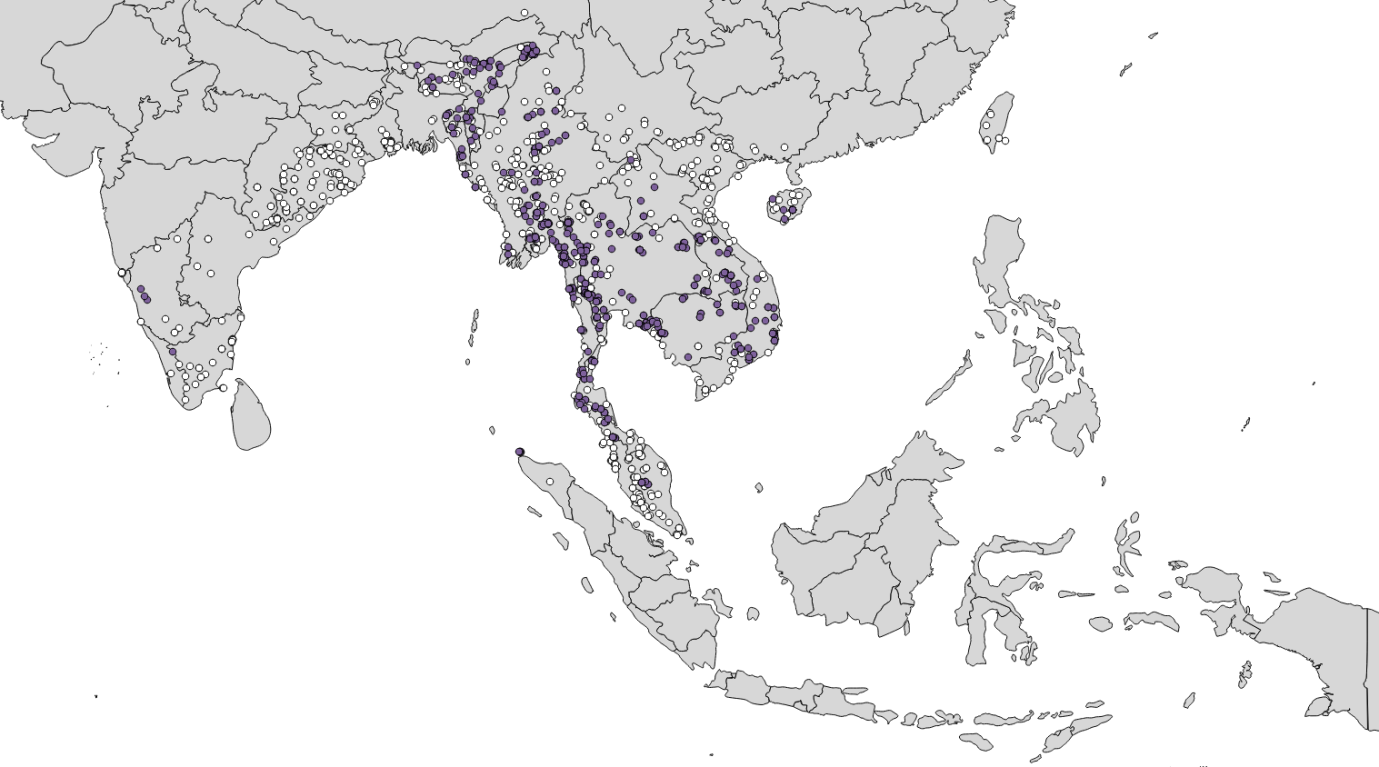
**

**Leucosphyrus Complex data**

**
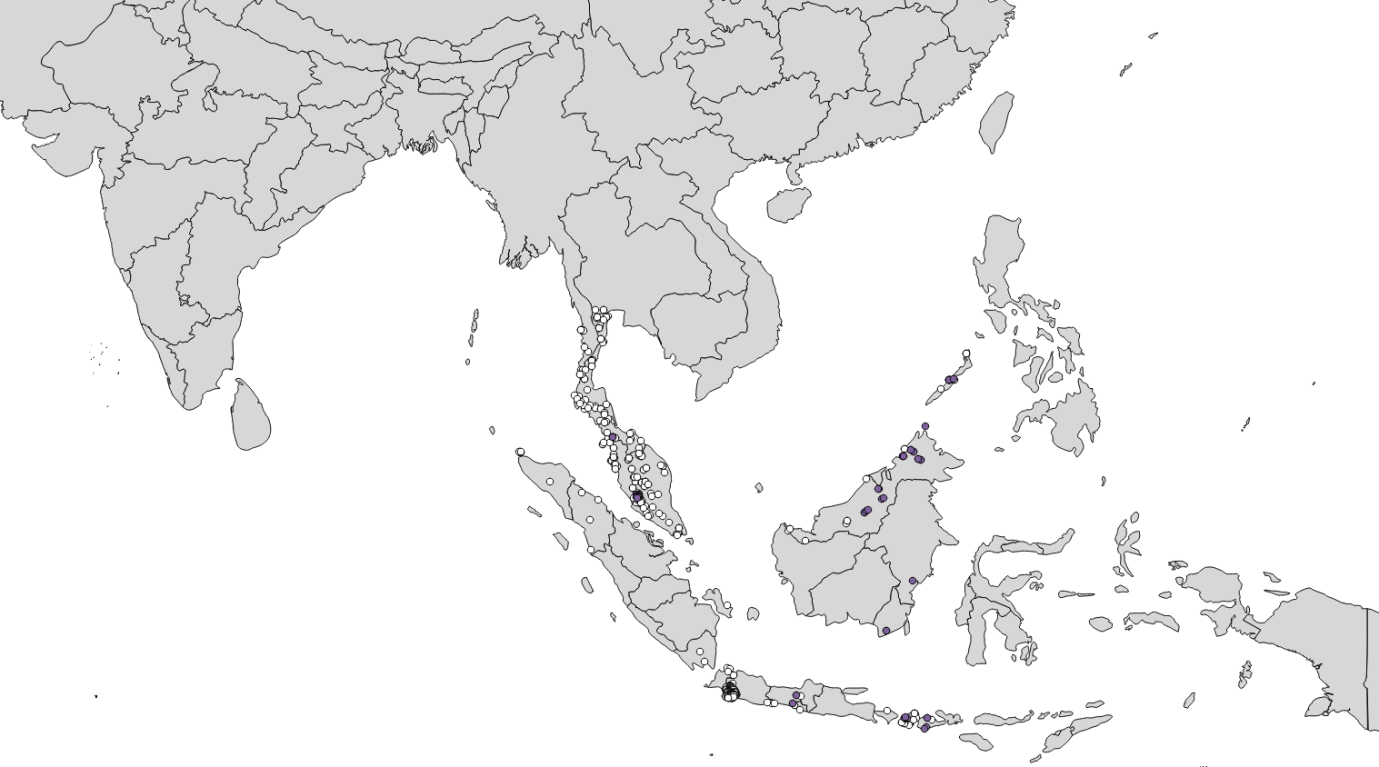
**

533

**Leucosphyrus Group data**

**
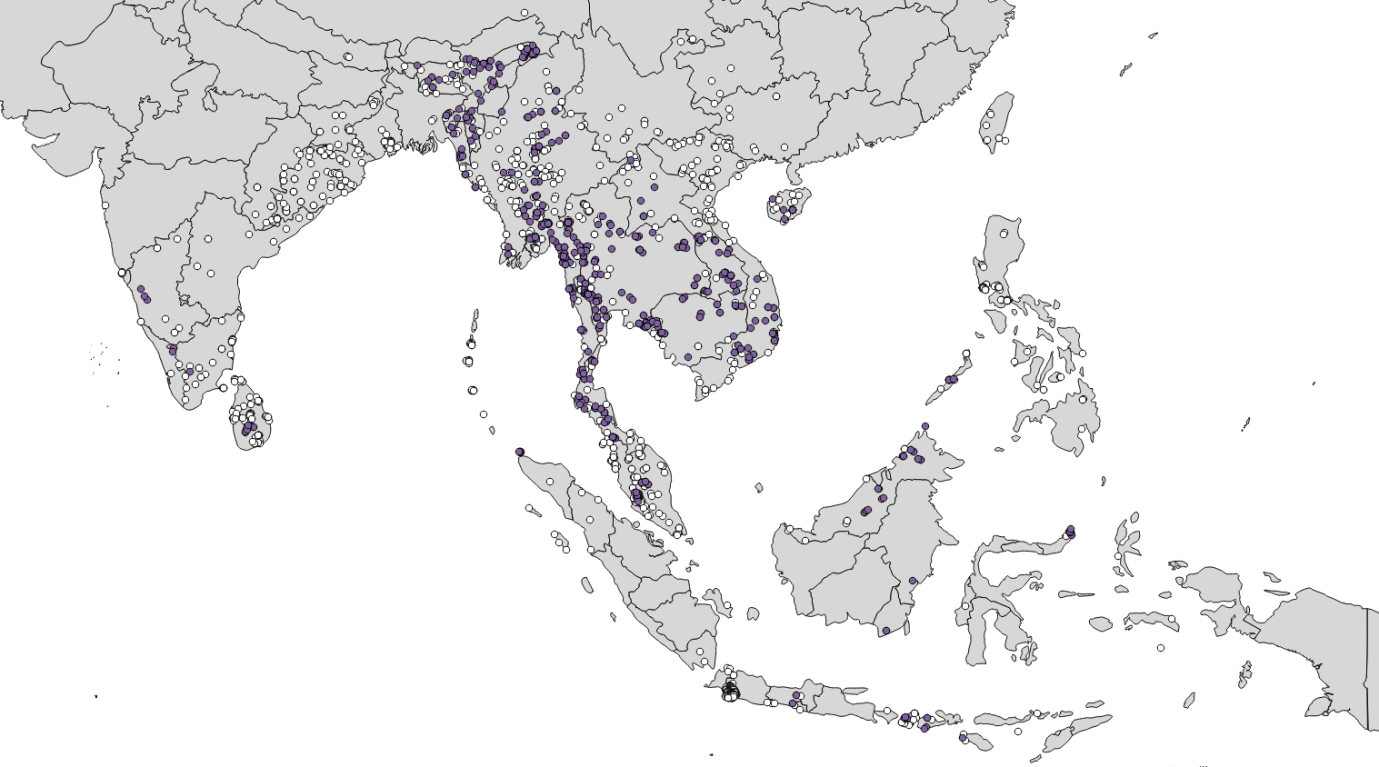
**

600
