# Supplementary material for: Predicting the geographical distributions of the macaque hosts and mosquito vectors of Plasmodium knowlesi malaria in forested and non-forested areas
Source: Parasit Vectors. 2016 Apr 28;9:242. doi: 10.1186/s13071-016-1527-0 (PMC4850754; doi:10.1186/s13071-016-1527-0)
Supplement: Additional file 2: — Environmental variables used in the species distribution models and construction of the forest cover layers. Details of the covariate data used are provided together with their source details. The construction and validation of the intact and disturbed forest layers is described in detail. (DOCX 27 kb) [file 13071_2016_1527_MOESM2_ESM.docx]

**Environmental variables used in the species distribution models and construction of the forest cover layers**

***Environmental variables used in the species distribution models***

| **Variable** | **Data source** |
| --- | --- |
| Enhanced vegetation index  (mean and standard deviation of the monthly values, 2000-14) | MODIS satellite [1] |
| Land surface temperature  (mean and standard deviation of the monthly values, 2000-14) | MODIS satellite [1] |
| Tasseled cap wetness  (mean and standard deviation of the monthly values, 2000-14) | MODIS satellite [1] |
| Tasseled cap brightness  (mean and standard deviation of the monthly values, 2000-14) | MODIS satellite [1] |
| Land cover classes (proportional)  for each year from 2001 to 2012 | MODIS satellite [1] |
| Elevation | Shuttle Radar Topography Mission |
| Intact forest cover (proportional)  for each year from 2001 to 2012 | MODIS satellite [1]  Intact Forest Landscape [2] |
| Disturbed forest cover (proportional)  for each year from 2001 to 2012 | MODIS satellite [1]  Intact Forest Landscape [2] |
| Human population density (2010) | WorldPop [3] Gridded Population of the World [4] |

The enhanced vegetation index provides a measure of greenness and moisture. Tasseled cap wetness is associated with surface moisture and tasseled cap brightness is associated with moisture in areas with bare soil or senescent vegetation.

The proportional cover of the following land classes for each year from 2001 to 2012 was used:

- Woody savannah (trees 30-60% and understory vegetation)
- Savannah (trees 10-30% and understory vegetation)
- Open shrubland (vegetation <2m tall and shrub canopy cover >60%)
- Grassland (herbaceous cover with trees/shrubs <10%)
- Wetlands (a permanent mixture of water and vegetation over extensive areas)
- Cropland (temporary crops with harvest period or bare soil)
- Cropland-natural vegetation mosaic (mosaic of cropland, forest, shrubland or grassland)

Forest cover is defined in the International Geosphere and Biosphere Programme (IGBP) land cover classification as canopy cover >60% and height > 2m. This classification was split into intact and disturbed forest cover as detailed in the following section.

1. MODIS online data repository, accessed 20/04/2014, <http://e4ftl01.cr.usgs.gov/MODIS_Composites/MOTA/MCD12Q1.051/2001.01.01/>.
2. Intact Forest Landscapes methodology web page, accessed 20/04/2014, <http://intactforests.org/world.methodology.html> .
3. WorldPop population data repository, accessed 06/07/2015, <http://www.worldpop.org.uk/data/get_data/>.
4. Gridded Population of the World spatially diaggregated populations layers, accessed 06/07/2015, <http://sedac.ciesin.columbia.edu/data/collection/gpw-v3/sets/browse>.

***Construction of the forest cover data layers***

Annual covariate data layers for intact and disturbed forest cover were constructed for this study because 1) forest cover in this region has changed substantially during the period of study and 2) we are interested in any differences in species occurrence between forests where humans spend time and those that are free from human activity.

We used satellite-derived data on forest cover provided by the MODIS Land Cover Type Product [1], which is available annually from 2001 and, therefore, addresses the issue of changing forest cover within our study period. The MODIS data, however, does not distinguish between forest types where human activity is likely to be minimal and forest types where human activity is likely to be frequent. To address this issue, we used the Intact Forest Landscapes (IFL) 2000 dataset [2] which provides data on the coverage of “intact forest”, defined as “‘an unbroken expanse of natural ecosystems within the zone of current forest extent, showing no signs of significant human activity and large enough that all native biodiversity, including viable populations of wide-ranging species, could be maintained”. These data have been generated for conservation purposes but the classification of forested areas with no significant human activity meets our needs.

In summary, we prepared binary and fractional data layers on forest cover, and classified this extent into two forest types; intact forest and disturbed forest.

*Preparation of annual binary forest cover layers derived from IGBP Land Cover data*

The IGBP Land Cover layer is one of several land cover classification products derived from MODIS satellite imagery, and it was selected for use in this application as it was the most diverse classification scheme available at multiple points in time for the study period. The annual temporal resolution of the IGBP land cover layer provides a means of assessing how forest types identified in the IFL dataset change from 2000 onward. For example, cells classified as intact forest in the IFL that transition from forest to alternative land cover types (e.g., cropland) in subsequent years, clearly no longer meet the definition of intact forest.

The MODIS data was acquired in tile format from the MODIS data repository [3] and then pre-processed for subsequent analysis. A total of 76 tiles per year were required to cover the study area and, once obtained, these tiles were converted into seamless annual mosaics using the MODIS Reprojection Tool (MRT). The five forest classes within the IGPB scheme (Evergreen Needleleaf forest, Evergreen Broadleaf forest, Deciduous Needleleaf forest, Deciduous Broadleaf forest, and Mixed forest) were then combined to create an annual IGBP forest layer coded as 1 (forest) and 0 (non-forest).

*Preparation of annual binary data layers derived from Intact Forest Landscapes data*

The Intact Forest Landscapes (IFL) 2000 dataset was obtained in shapefile format from the IFL website [2] and used as the baseline to generate annual intact forest surfaces. The IFL 2005 and IFL 2010 datasets were only available for Indonesia and Papua New Guinea so they were not used to create covariate layers for the niche model, but were used in the data validation steps described below. The 2000 dataset was clipped to our study area and converted to a 500m resolution raster surface with value ‘1’ for each intact forest area and ‘0’ for elsewhere, that was configured to match the IGBP data (using extent 60.04161865N, -49.999908W, 179.04152305E, -20.000016S).

To create annual intact forest data, first the extent of cells classified as forest from IGBP 2001 that intersected intact forest data from IFL 2000 was extracted. This step was then repeated using the data from IGBP for each year combined with the intact forest layer generated for the previous year, to derive annual intact forest layers up to 2012. Using intact forest from the preceding year, as opposed to the original 2000 IFL dataset, prevented any regrowth of forest from being considered as intact forest.

*Preparation of annual binary data layers representing disturbed forest cover*

Areas of forest that are not considered “intact forest” include timber concessions, rubber plantations, palm plantations, mining concessions and so on. These areas are of relevance to the *P. knowlesi* mapping work and were grouped together in one class that we have called “disturbed forest”. This extent was created by subtracting the intact forest binary layer from the IGBP forest extent binary layer, in each year.

*Creation of fractional data layers for all three data types*

The resulting forest datasets (intact, disturbed, and original IGPB) were then converted from 500m spatial resolution to the 5km analysis resolution utilised in the subsequent analyses. This conversion process involved summing the areal portions of all 500m cells falling within each 5km output cell, and then dividing by the area of the output cell, to produce fractional forest layers for each dataset for each year. The resulting products effectively converted the 500m forest rasters from categorical to continuous data, with all cell values in the output products ranging from 0.0 (no forest) to 1.0 (100% forest covered).

*Validation of annual forest cover data*

The data used to define forest cover were derived from satellite data, and the data used to sub-divide forest cover into intact forest and disturbed forest classes came from multiple sources, including transport maps, satellite data, and by using visual interpretation [4]. No system that infers land class is perfect and so we validated the data layers generated above using alternative data sources. These data sources came in a variety of formats so an appropriate validation method was selected individually for each alternative data source.

*Validation using IFL 2005 and 2010 data for Indonesia and Papua New Guinea*

IFL 2005 and IFL 2010 datasets available for Indonesia and Papua New Guinea [2] were used to validate the intact forest layers generated for these two years at the two locations. Accuracy assessment [5,6] was carried out to validate the binary surfaces (Table 1). The r^2^ and Root Mean Square Error values were calculated to evaluate the relationship between the fractional surfaces (Table 2).

Table 1. Binary intact forest layer validation by accuracy assessment

| **Year** | **Overall Accuracy** | **Kappa Coefficient** |
| --- | --- | --- |
| **2005** | 0.97138 | 0.91425 |
| **2010** | 0.95566 | 0.86250 |

Table 2. Fractional intact forest layer validation

|  | **All areas included** | | **Intact forest areas only** | |
| --- | --- | --- | --- | --- |
| **Year** | **r^2^** | **RMSE** | **r^2^** | **RMSE** |
| **2005** | 0.91321 | 0.11037 | 0.71899 | 0.10981 |
| **2010** | 0.83646 | 0.14881 | 0.54696 | 0.26448 |

*Validation using available concession and plantation datasets*

Multiple sources of concession data in Indonesia and Cambodia, and plantation data in Peninsular Malaysia were used for validation. These data were all available in shapefile format. The validation process was conducted by using the ‘iscetpolyrst’ (Intersect Polygons With Raster) function in the Geospatial Modelling Environment (GME) tool to assess the percentage of land cover types falling in these concession and plantation areas.

*Concession data from Indonesia*

Concession data including logging concessions, oil palm concessions and timber concessions were obtained from Global Forest Watch [7]. The validation tests were conducted on each concession type individually and on all types combined. The concession dataset was not linked to a specific year so we compared it to our layers for both 2001 and 2012.

Greater than 67% of the concession areas overlapped with our disturbed forest layer (Table 3). A few oil palm concessions (less than 3%, Table 4) and timber concessions (less than 7%, Table 5) fell within our intact forest layer, whereas about a quarter of logging concessions were identified within our intact forest layer (Table 6).

Table 3. The iscetpolyrst statistic for all concession data

| **Year** | **Disturbed forest layer** | **Intact forest layer** | **Other land** |
| --- | --- | --- | --- |
| **2001** | 67.44% | 16.12% | 16.44% |
| **2012** | 69.57% | 15.06% | 15.37% |

Table 4. The iscetpolyrst statistic for oil palm concession data

| **Year** | **Disturbed forest layer** | **Intact forest layer** | **Other land** |
| --- | --- | --- | --- |
| **2001** | 67.48% | 2.74% | 29.78% |
| **2012** | 66.86% | 2.30% | 30.85% |

Table 5. The iscetpolyrst statistic for timber concession data

| **Year** | **Disturbed forest layer** | **Intact forest layer** | **Other land** |
| --- | --- | --- | --- |
| **2001** | 69.50% | 6.99% | 23.51% |
| **2012** | 72.49% | 5.94% | 21.56% |

Table 6. The iscetpolyrst statistic for logging concession data

| **Year** | **Disturbed forest layer** | **Intact forest layer** | **Other land** |
| --- | --- | --- | --- |
| **2001** | 67.37% | 26.48% | 6.15% |
| **2012** | 70.51% | 25.05% | 4.43% |

*Concession data from Cambodia*

The Economic Land Concession (ELC) and mining concession data in Cambodia were both obtained from Open Development [8]. Neither dataset is linked to forest areas specifically, so we did not expect a strong link with our disturbed forest layers, however, we did expect a negative relationship with our intact forest layers. The validation using ELC data was implemented for each year available. The mining concession dataset was not linked to a specific year so we evaluated it against our layers for years 2001 and 2012.

Most of the ELC areas overlapped with non-forest land in our classification. The majority of the remaining ELC areas fell within our disturbed forest areas and very few were in our intact forest areas (0.15% in 2010 and 0% in other years, Table 7). Similarly, very few mining concessions were found in our intact forest areas (less than 0.06%, Table 8).

Table 7. The iscetpolyrst statistic for Economic Land Concession data, by year

| **Year** | **Disturbed forest layer** | **Intact forest layer** | **Other land** |
| --- | --- | --- | --- |
| **2001** | 20.97% | 0% | 79.03% |
| **2004** | 8.36% | 0% | 91.64% |
| **2005** | 33.38% | 0% | 66.62% |
| **2006** | 35.90% | 0% | 64.10% |
| **2007** | 48.53% | 0% | 51.47% |
| **2008** | 50.63% | 0% | 49.37% |
| **2009** | 12.97% | 0% | 87.03% |
| **2010** | 49.89% | 0.15% | 49.97% |
| **2011** | 46.26% | 0% | 53.74% |
| **2012** | 10.66% | 0% | 89.34% |

Table 8. The iscetpolyrst statistic for mining concession data

| **Year** | **Disturbed forest layer** | **Intact forest layer** | **Other land** |
| --- | --- | --- | --- |
| **2001** | 50.95% | 0.044% | 49.01% |
| **2012** | 42.14% | 0.052% | 57.81% |

*Plantation data from Peninsular Malaysia*

Data for rubber plantation cover and oil palm plantation cover were obtained from the Malaysia Geoportal [9], which provides the location of rubber and oil palms planted in Peninsular Malaysia. The data layer was published in 2010. It was originally based on 2004 data and updated as needed by the Malaysian authorities. The validation was carried out on our data layers for the years 2004 and 2010.

Approximately 42% of the rubber plantations were found in our disturbed forest areas in 2004 and this figure went up to 56% in 2010, while no rubber plantations were found in our intact forest areas in either year (Table 9). Oil palm plantations were found chiefly in our disturbed forest areas with 77.65% in 2004 and 77.01% in 2010, and less than 5% overlapped with our intact forest layer (Table 10).

Table 9. The iscetpolyrst statistic for rubber plantation data

| **Year** | **Disturbed forest layer** | **Intact forest layer** | **Other land** |
| --- | --- | --- | --- |
| **2004** | 41.84% | 0% | 58.16% |
| **2010** | 56.46% | 0% | 43.54% |

Table 10. The iscetpolyrst statistic for oil palm plantation data

| **Year** | **Disturbed forest layer** | **Intact forest layer** | **Other land** |
| --- | --- | --- | --- |
| **2004** | 77.65% | 4.80% | 17.55% |
| **2010** | 77.01% | 4.80% | 18.19% |

*References*

1. Online user guide for the MODIS Land Cover Type Product, accessed 20/04/2014 (http://www.bu.edu/lcsc/files/2012/08/MCD12Q1_user_guide.pdf)
2. Intact Forest Landscapes website, accessed 20/04/2014 (http://intactforests.org/)
3. MODIS online data repository, accessed 20/04/2014 <http://e4ftl01.cr.usgs.gov/MODIS_Composites/MOTA/MCD12Q1.051/2001.01.01/>
4. Intact Forest Landscapes methodology web page, accessed 20/04/2014 http://intactforests.org/world.methodology.html
5. Horning, N. & Nelson, R. 2008 Overview of accuracy assessment of land cover products. American Museum of Natural History, New York.
6. Congalton, R.G. 1991 A review of assessing the accuracy of classifications of remotely sensed data. *Remote Sens. Environ.* **37**, 35-46. (doi:10.1016/0034-4257(91)90048-b).
7. Global Forest Watch website, accessed 07/05/2014 ([www.globalforestwatch.org](http://www.globalforestwatch.org/))
8. Open Development website, accessed 09/05/2014 (<http://www.opendevelopmentcambodia.net/>)
9. Malaysia Geoportal plantation data website, accessed 15/05/2014 (http://mygdix.mygeoportal.gov.my/mygdiexplorer/catalog/search/viewMetadataDetails.page?uuid=%7B48A834D7-1A83-498F-9B7A-E5B9A4DCDE9D%7D)
