# Supplementary material for: Predicting the geographical distributions of the macaque hosts and mosquito vectors of Plasmodium knowlesi malaria in forested and non-forested areas
Source: Parasit Vectors. 2016 Apr 28;9:242. doi: 10.1186/s13071-016-1527-0 (PMC4850754; doi:10.1186/s13071-016-1527-0)
Supplement: Additional file 3: — Investigating the impact of using annual land cover data. The model was run using identical datasets and either 1) year-matched land cover data or 2) 2012 land cover data. The resulting distributions are shown with the AUC ± standard error, and the top predictors with their relative influences. A map showing the difference between the two resulting distributions is also provided. (DOCX 2977 kb) [file 13071_2016_1527_MOESM3_ESM.docx]

**Investigating the impact of using annual land cover data**

The model was run using identical datasets and either 1) year-matched land cover data or 2) 2012 land cover data. The resulting distributions are shown with the AUC ± standard error, and the top predictors with their relative influences. A map showing the difference between the two resulting distributions is also provided.


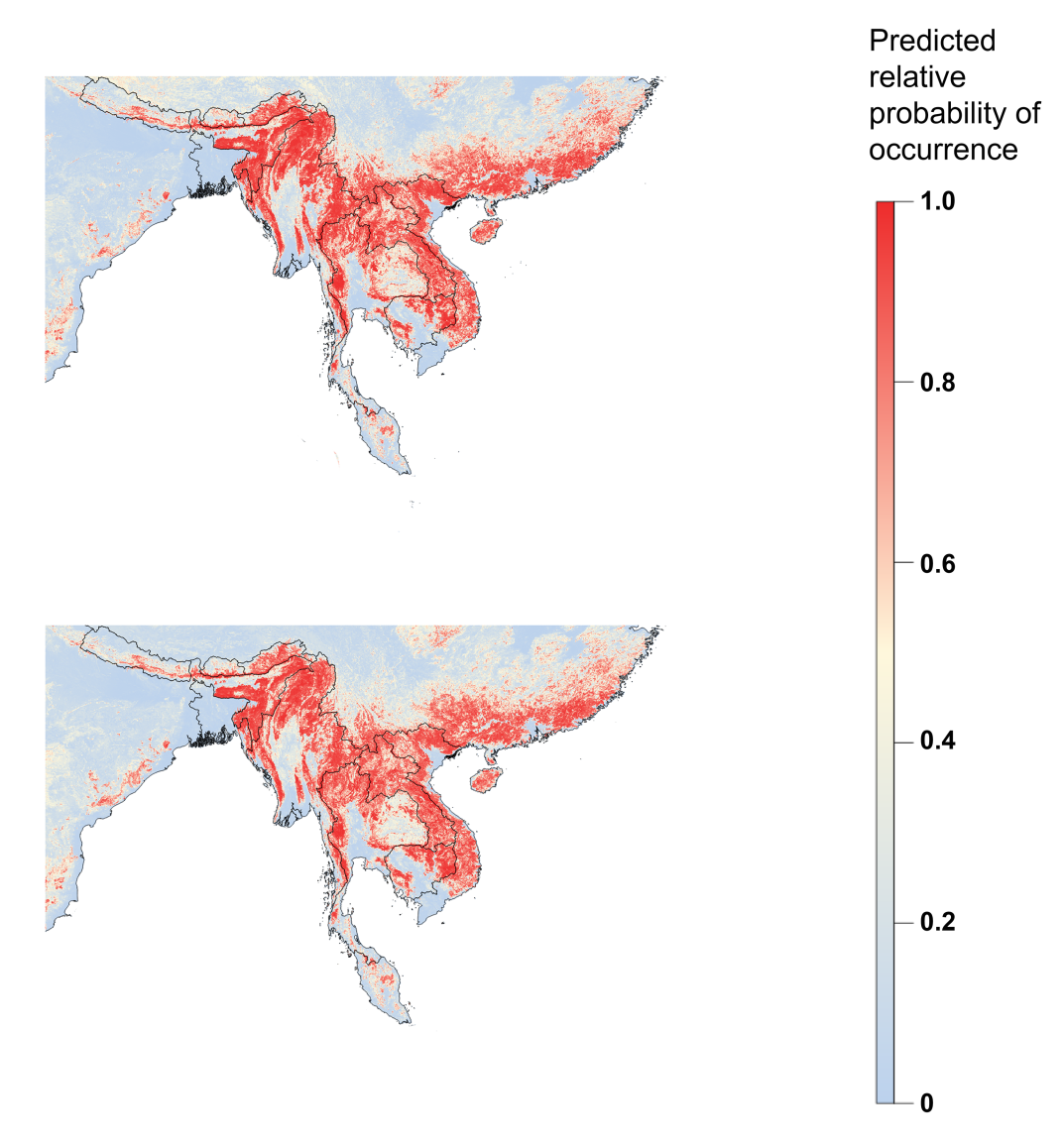
***Macaca leonina***

**Using annual land cover data**

AUC = 0.835 ± 0.002

***Top predictors*^1^**

Elevation 27.24

Enhanced veg. index 10.03

Seasonality veg. index 7.62

Disturbed forest 6.67

Human pop. 6.13

Seasonality in moisture 5.64

**Using 2012 land cover data**

AUC = 0.783 ± 0.002

***Top predictors*^1^**

Elevation 28.67

Enhanced veg. index 10.36

Seasonality veg. index 7.68

Human pop. density 6.69

Seasonality in moisture 6.14

Moisture 5.70

Woody savannah 5.28

**The difference between the values in the two distributions above**


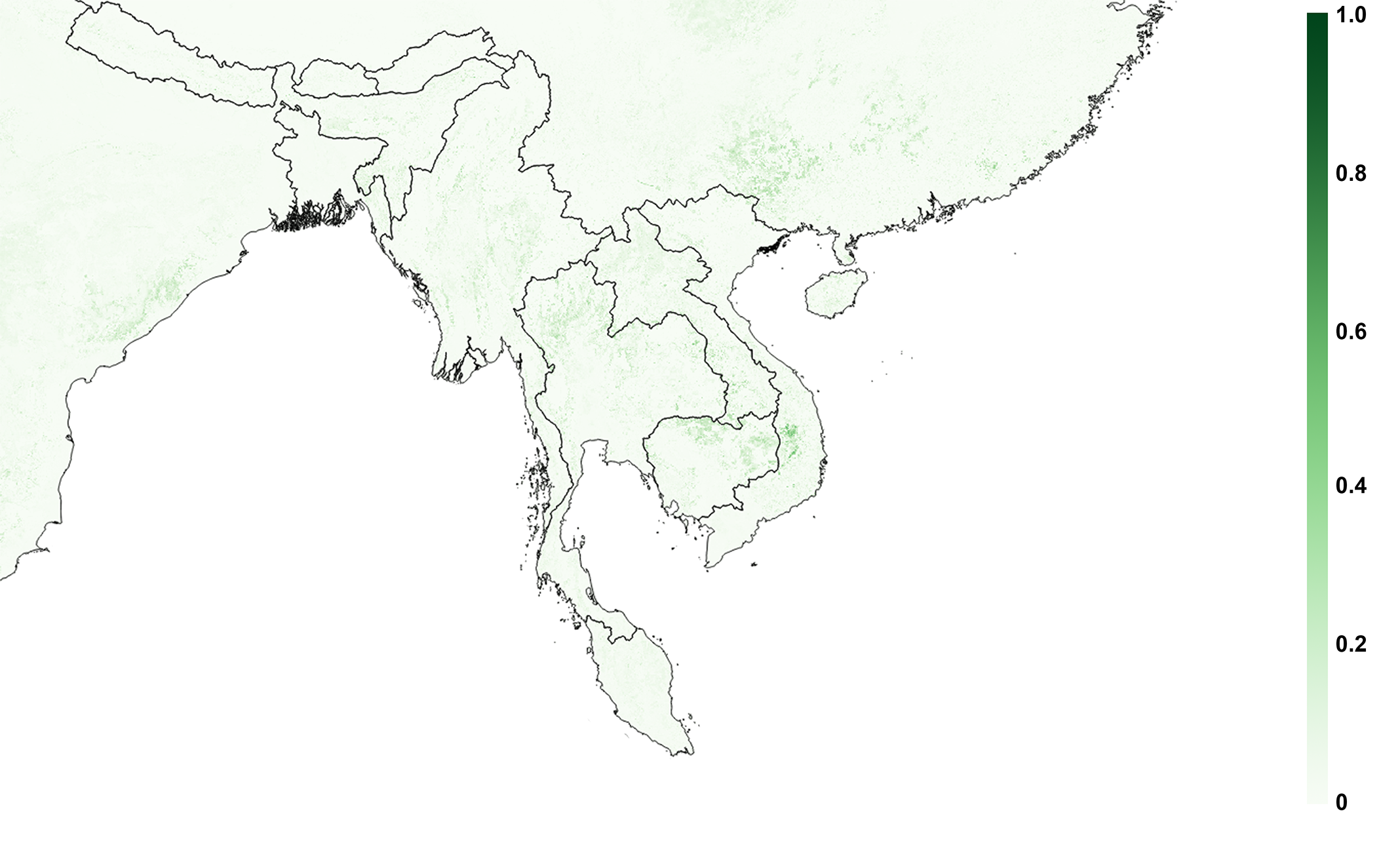


**Dirus Complex**


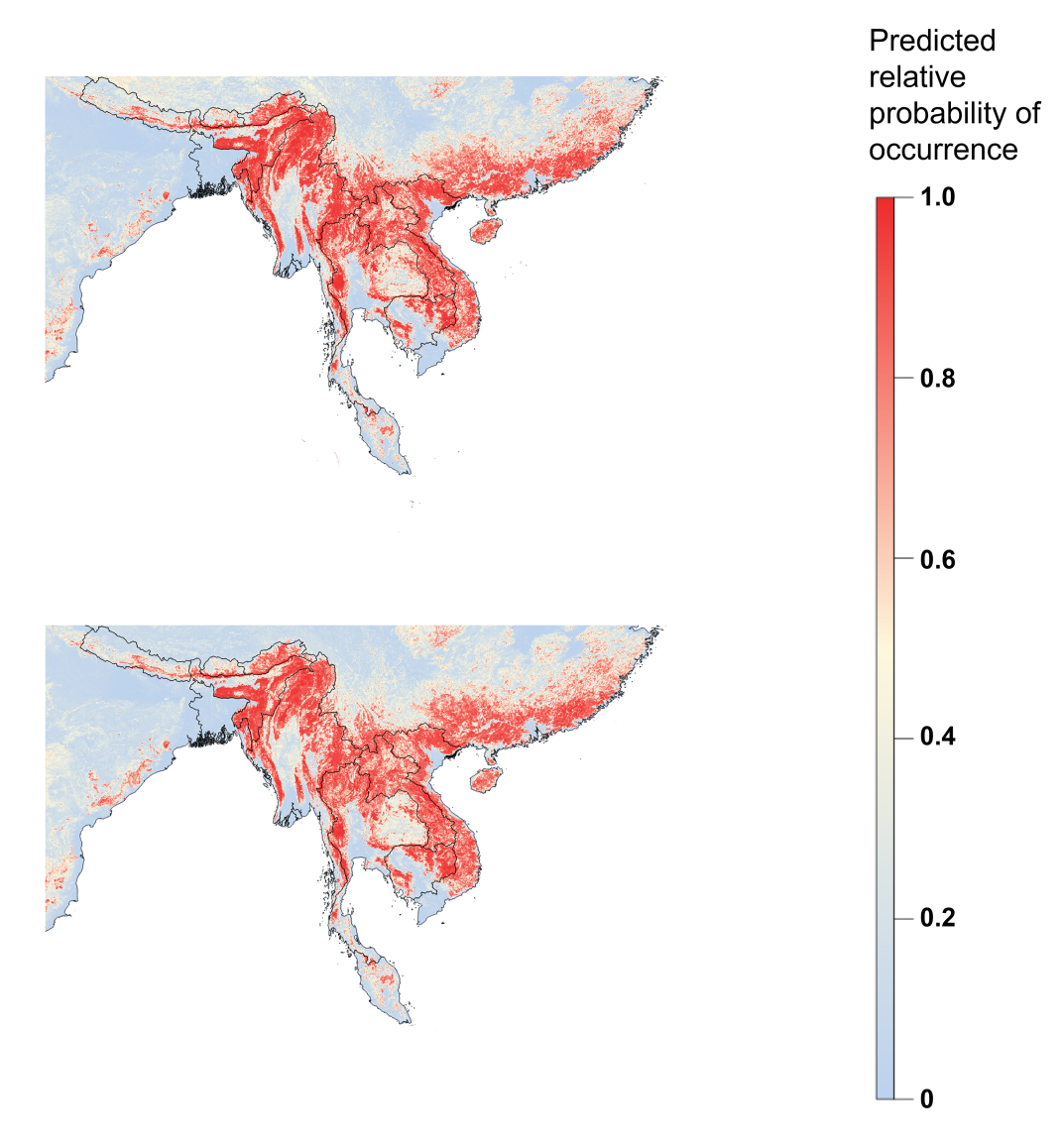
**Using annual land cover data**

AUC = 0.833 ± 0.003

***Top predictors*^1^**

Human pop. 22.38

Enhanced veg. index 12.32

Seasonality veg. index 10.34

Seasonality in temp. 8.95

Daytime temperature 7.58

Seasonality in moisture 6.58

Moisture 5.62

**Using 2012 land cover data**

AUC = 0.828 ± 0.004

***Top predictors*^1^**

Human pop. 24.00

Enhanced veg. index 12.41

Seasonality veg. index 10.20

Seasonality in temp. 8.87

Daytime temperature 7.50

Seasonality in moisture 6.56

Woody savannah 5.85

**The difference between the values in the two distributions above**


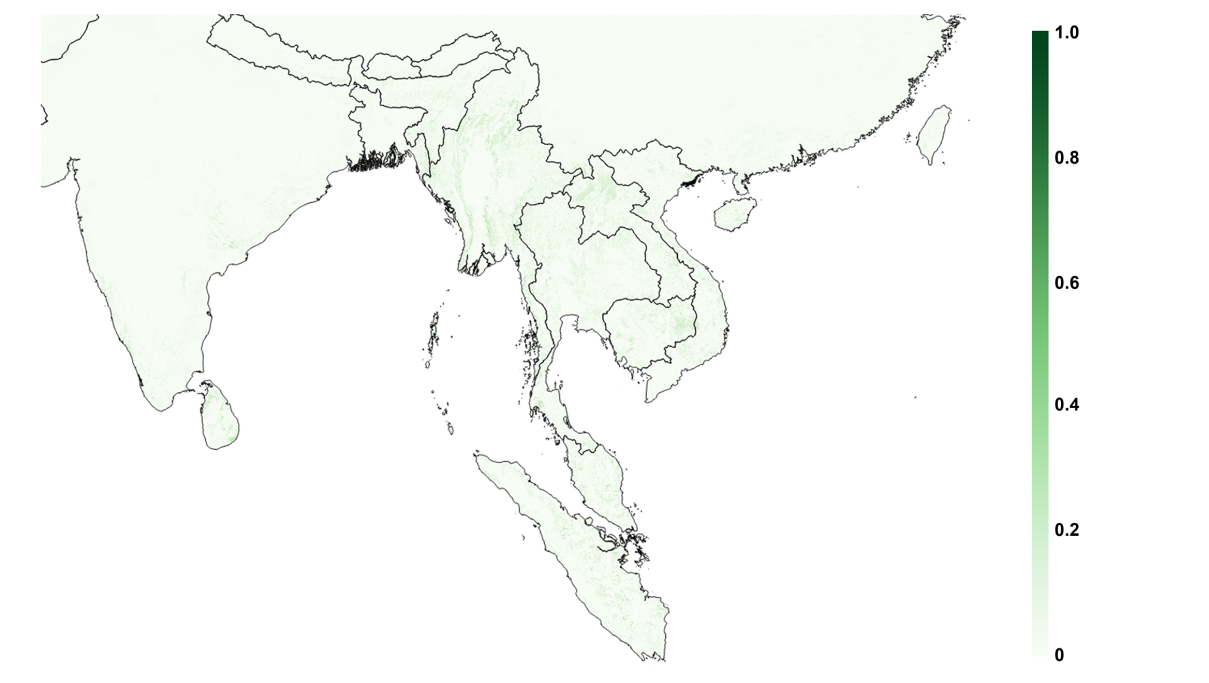


1. Full descriptions of the predictors are given in Supplementary File 2.
