# Supplementary material for: Predicting the geographical distributions of the macaque hosts and mosquito vectors of Plasmodium knowlesi malaria in forested and non-forested areas
Source: Parasit Vectors. 2016 Apr 28;9:242. doi: 10.1186/s13071-016-1527-0 (PMC4850754; doi:10.1186/s13071-016-1527-0)

**The 0.025 and 0.975 quantile model predictions, and the top predictors, for each macaque species**

A total of 19 predictors were tested in each model and the sum of their relative influence values is 100. The top predictors are defined as those whose relative influence was greater than 100/19, i.e. the value that would be expected if no predictors were better than any other. The relative influence values for the top predictors are shown in the tables below.

***Macaca fascicularis***

| **Predictor** | **Relative influence** |
| --- | --- |
| Disturbed forest cover | 14.93 |
| Elevation | 11.98 |
| Human population density | 10.64 |
| Daytime temperature | 9.57 |
| Permanent wetland cover | 8.66 |
| Temporal variation in daytime temperature | 7.29 |
| Temporal variation in tasseled cap brightness (seasonality in moisture) | 6.11 |
| Temporal variation in tasseled cap wetness (seasonality in moisture) | 5.47 |


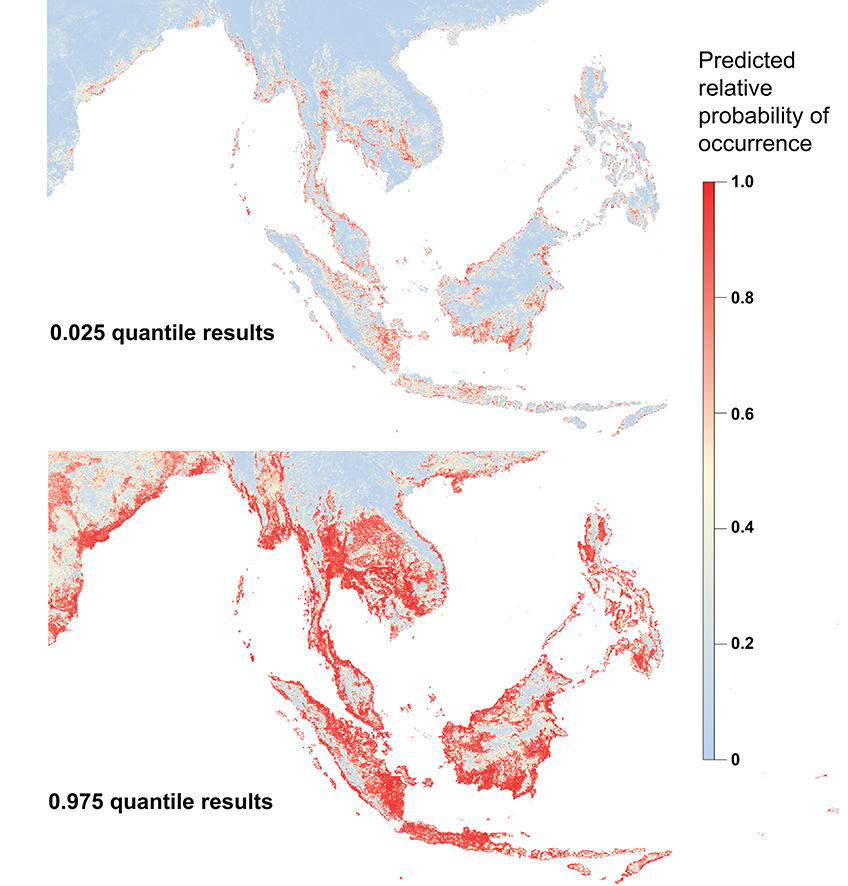
***Macaca nemestrina***

| **Predictor** | **Relative influence** |
| --- | --- |
| Enhanced vegetation index (greenness and moisture) | 30.10 |
| Daytime temperature | 13.92 |
| Human population density | 10.54 |
| Temporal variation in daytime temperature | 8.92 |
| Cropland-natural vegetation mosaic cover | 7.69 |
| Permanent wetland cover | 6.36 |


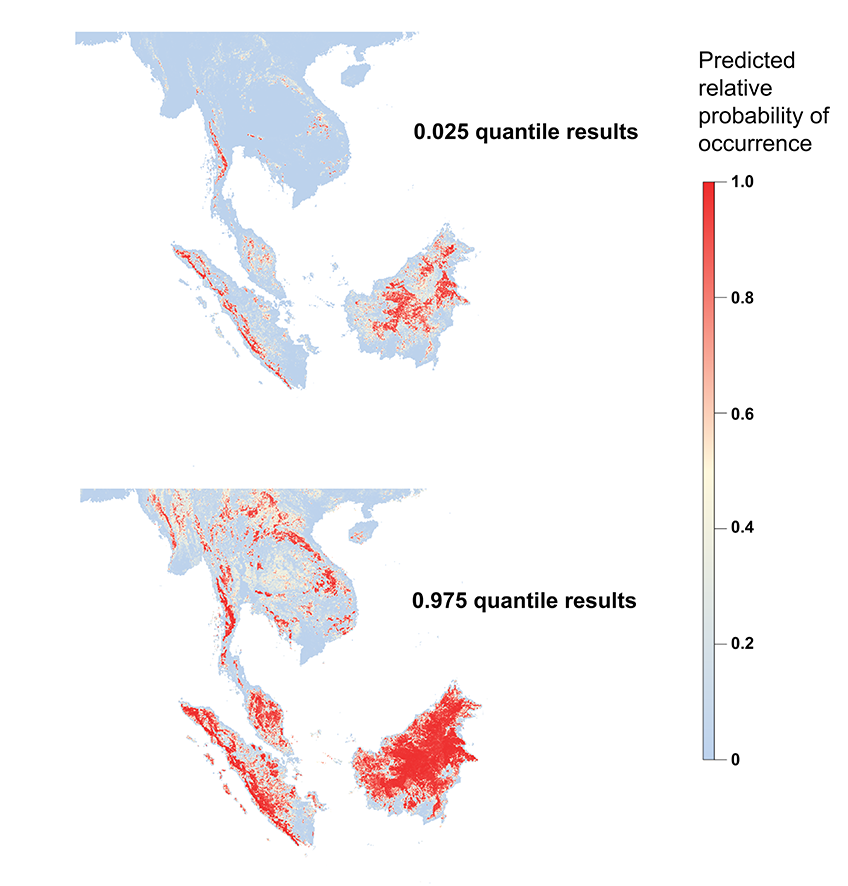


***Macaca leonina***

| **Predictor** | **Relative influence** |
| --- | --- |
| Elevation | 23.12 |
| Temporal variation in tasseled cap wetness (seasonality in moisture) | 9.58 |
| Woody savannah (30-60% tree canopy) cover | 7.99 |
| Enhanced vegetation index (greenness and moisture) | 7.34 |
| Tasseled cap brightness (moisture) | 6.83 |
| Temporal variation in the enhanced vegetation index (seasonality in greenness/moisture) | 6.37 |
| Human population density | 5.92 |
| Temporal variation in daytime temperature | 5.47 |


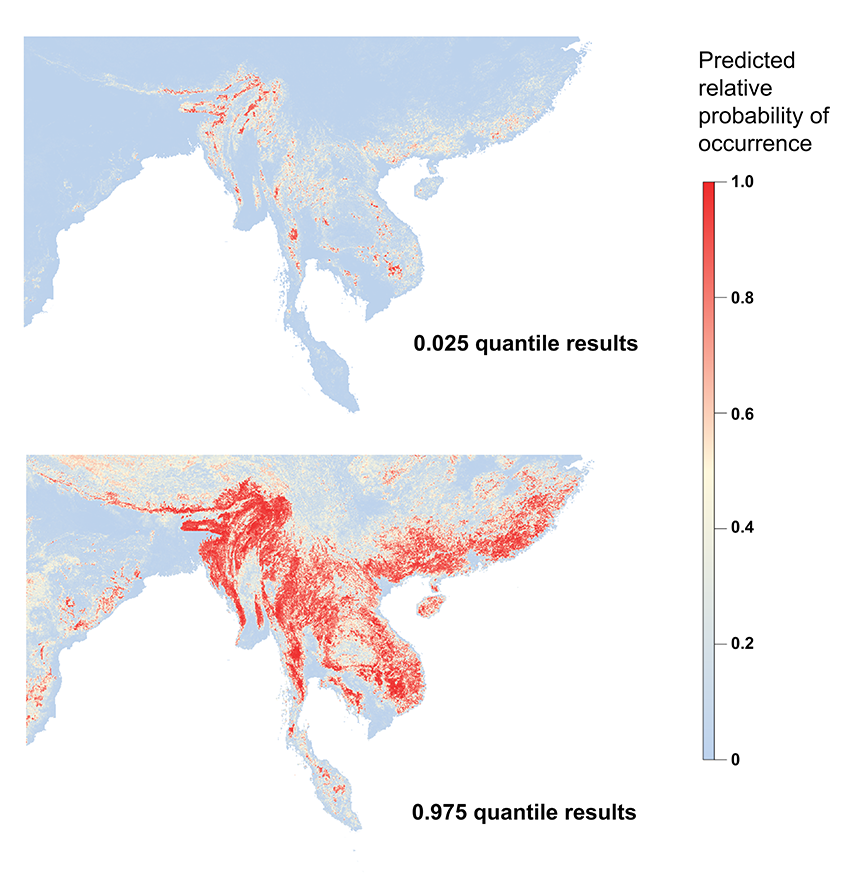

Supplement: Additional file 4: — The 0.025 and 0.975 quantile model predictions, and the top predictors, for each macaque species. For each macaque species the 0.025 and 0.975 quantile model outputs, masked out on islands outside each species range, are provided with the mean AUC (± standard error) and the relative influence of the top predictors for that species. (DOCX 1183 kb) [file 13071_2016_1527_MOESM4_ESM.docx]
