# Supplementary material for: Predicting the geographical distributions of the macaque hosts and mosquito vectors of Plasmodium knowlesi malaria in forested and non-forested areas
Source: Parasit Vectors. 2016 Apr 28;9:242. doi: 10.1186/s13071-016-1527-0 (PMC4850754; doi:10.1186/s13071-016-1527-0)

**The 0.025 and 0.975 quantile model predictions, and the top predictors, for each mosquito model**

A total of 19 predictors were tested in each model and the sum of their relative influence values is 100. The top predictors are defined as those whose relative influence was greater than 100/19, i.e. the value that would be expected if no predictors were better than any other. The relative influence values for the top predictors are shown in the tables below.

***Anopheles dirus***

| **Predictor** | **Relative influence** |
| --- | --- |
| Temporal variation in daytime temperature | 16.28 |
| Tasseled cap brightness | 12.23 |
| Elevation | 10.46 |
| Human population density | 8.96 |
| Cropland cover | 7.86 |
| Enhanced vegetation index (greenness and moisture) | 6.91 |
| Temporal variation in tasseled cap brightness | 6.23 |
| Daytime temperature | 5.55 |


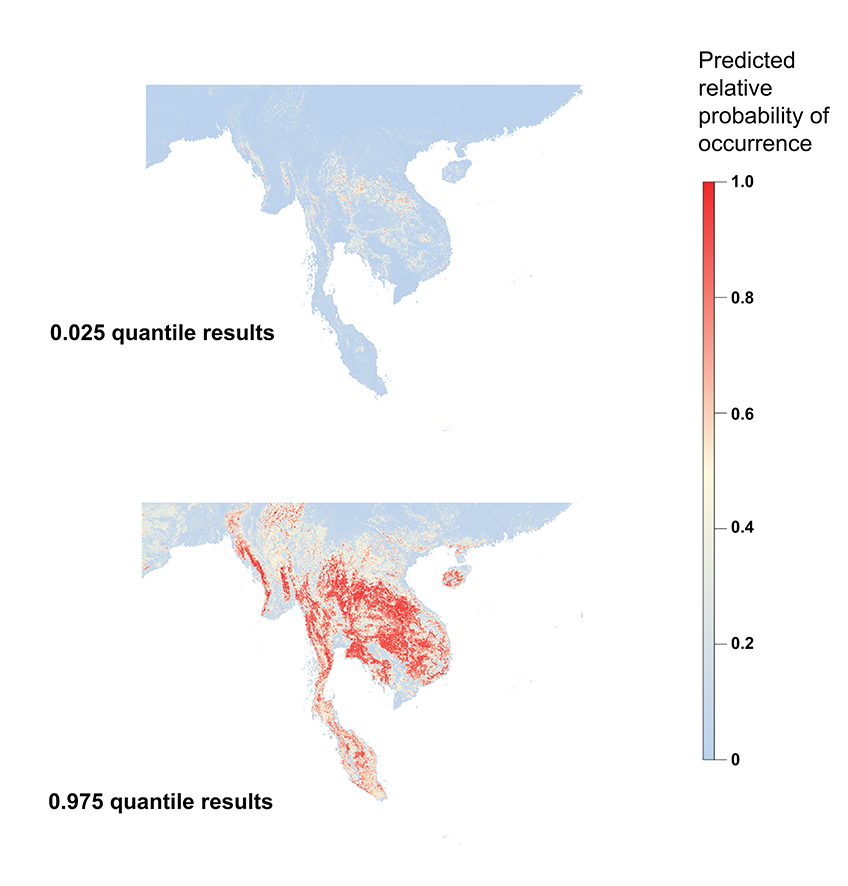


**Dirus Complex**

| **Predictor** | **Relative influence** |
| --- | --- |
| Temporal variation in daytime temperature | 15.30 |
| Enhanced vegetation index (greenness and moisture) | 10.95 |
| Human population density | 8.69 |
| Savannah cover | 7.75 |
| Temporal variation in the enhanced vegetation index (greenness/moisture) | 7.23 |
| Elevation | 6.70 |
| Temporal variation in tasseled cap brightness | 6.29 |
| Daytime temperature | 5.87 |
| Cropland cover | 5.50 |


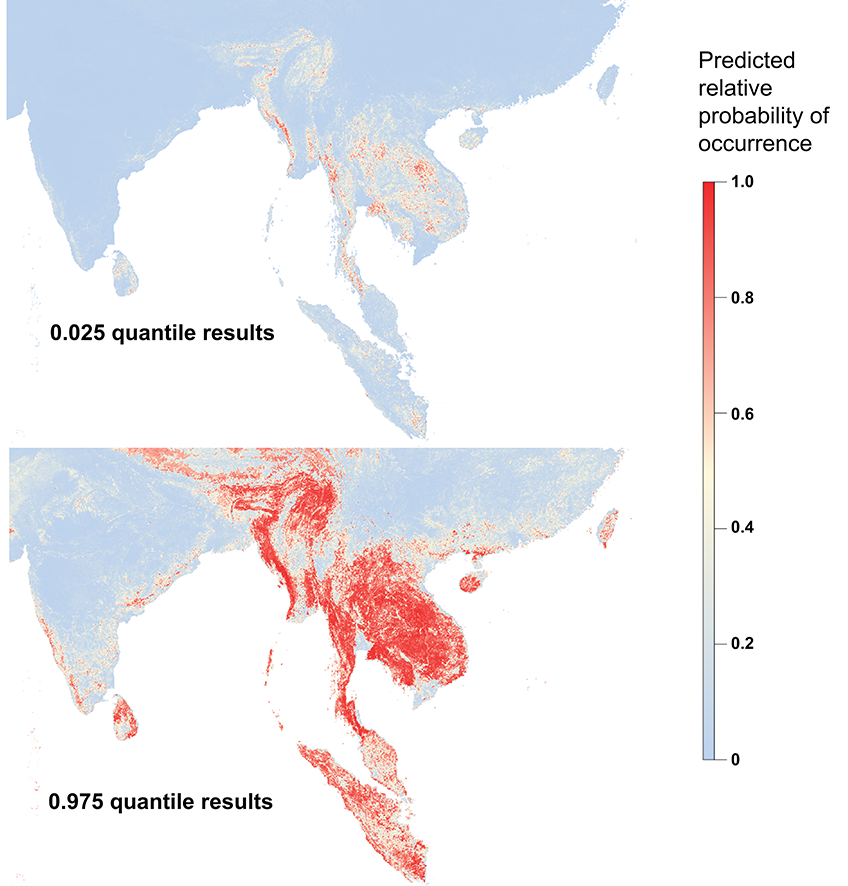


**Leucosphyrus Complex**

| **Predictor** | **Relative influence** |
| --- | --- |
| Human population density | 39.79 |
| Savannah land cover | 17.13 |
| Disturbed forest land cover | 11.19 |
| Temporal variation in daytime temperature | 8.16 |
| Tasseled cap brightness | 6.01 |


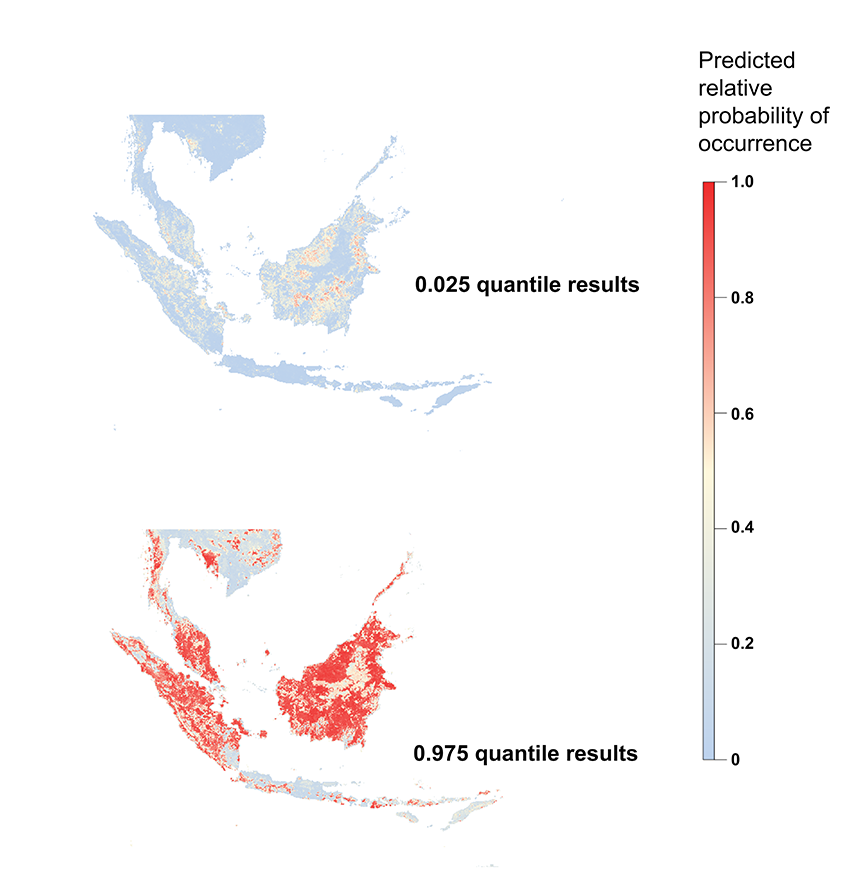


**Leucosphyrus Group**

| **Predictor** | **Relative influence** |
| --- | --- |
| Human population density | 26.16 |
| Tasseled cap wetness | 14.14 |
| Elevation | 9.37 |
| Temporal variation in daytime temperature | 7.37 |
| Temporal variation in the enhanced vegetation index (greenness/moisture) | 5.86 |


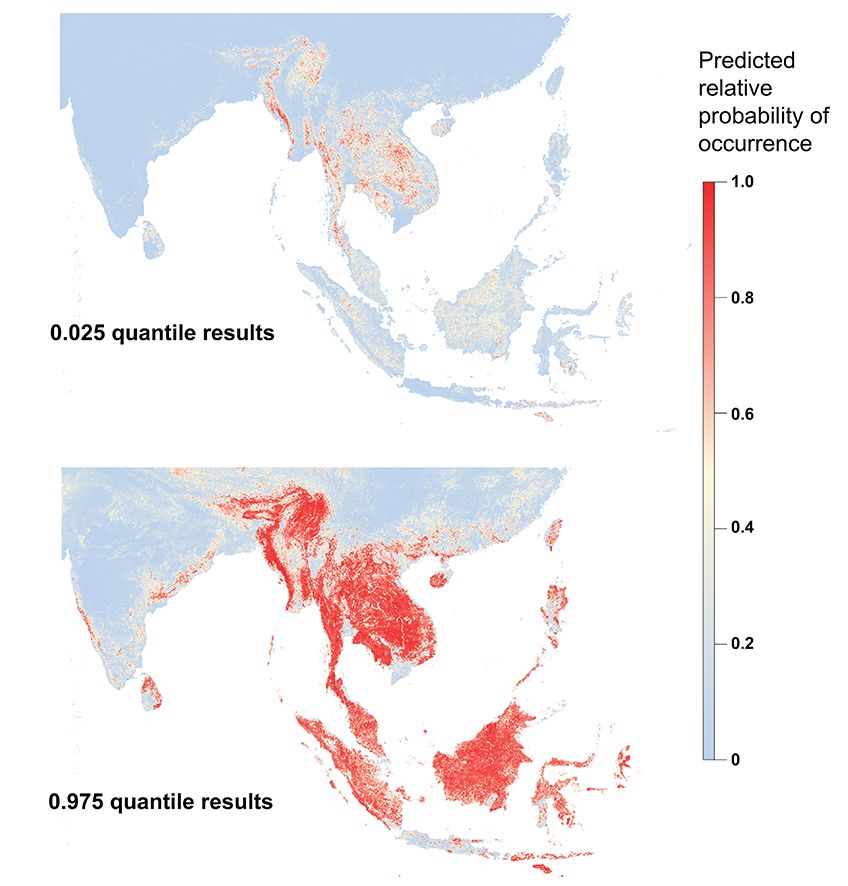

Supplement: Additional file 5: — The 0.025 and 0.975 quantile model predictions, and the top predictors, for each mosquito model. For each mosquito species, complex or group, the 0.025 and 0.975 quantile model outputs, masked out on islands outside each species or complex range, are provided with the mean AUC (± standard error) and the relative influence of the top predictors for that model. (DOCX 1331 kb) [file 13071_2016_1527_MOESM5_ESM.docx]
