# Supplementary material for: Predicting the geographical distributions of the macaque hosts and mosquito vectors of Plasmodium knowlesi malaria in forested and non-forested areas
Source: Parasit Vectors. 2016 Apr 28;9:242. doi: 10.1186/s13071-016-1527-0 (PMC4850754; doi:10.1186/s13071-016-1527-0)

**Proportional land cover in areas with high predicted probability of species occurrence**

***Land cover class definitions used for the density plots***

| Intact forest | Canopy cover >60% of the area and height exceeding 2m, with no signs of human activity and having an area of at least 500km^2^. |
| --- | --- |
| Disturbed forest | Canopy cover >60% of the area and height exceeding 2m with signs of human activity and/or broken cover. This class includes mature plantations, and timber and mining concessions. |
| Woody savannah | Canopy cover between 30-60% of the area and height exceeding 2m with understory plants. |
| Savannah | Canopy cover between 10-30% of the area and height exceeding 2m with understory plants. |
| Open shrubland | Woody vegetation less than 2m tall with cover between 10-60% of the area. |
| Cropland/natural vegetation mosaic | Mosaics of cropland, forest, shrubland or grassland. |
| Cropland | Temporary crops followed by a harvest period or bare soil. |
| Permanent wetland | A permanent mixture of water and herbaceous or woody vegetation. |
| Grassland | Herbaceous plants with <10% tree and shrub cover. |
| Urban | Land covered by buildings and other man-made structures. |

***Density plots***

The following plots (two pages) show the relative density of pixels at each percentage land class coverage for pixels where the probability of species occurrence was greater than 0.75. The density values were calculated from the ratio of pixels in the study area where the probability of species occurrence was greater than 0.75 to all pixels in the study area for that land class.


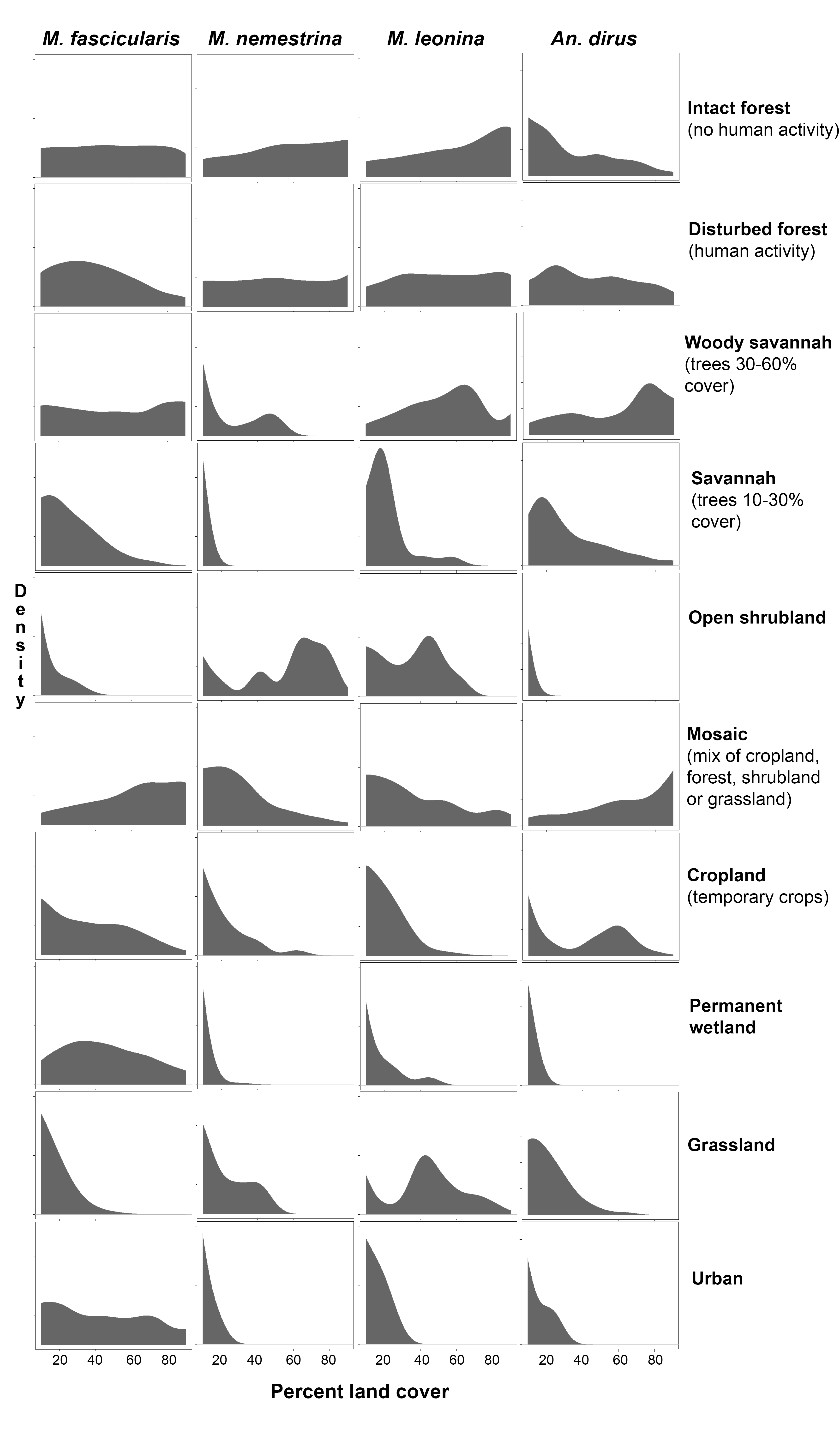


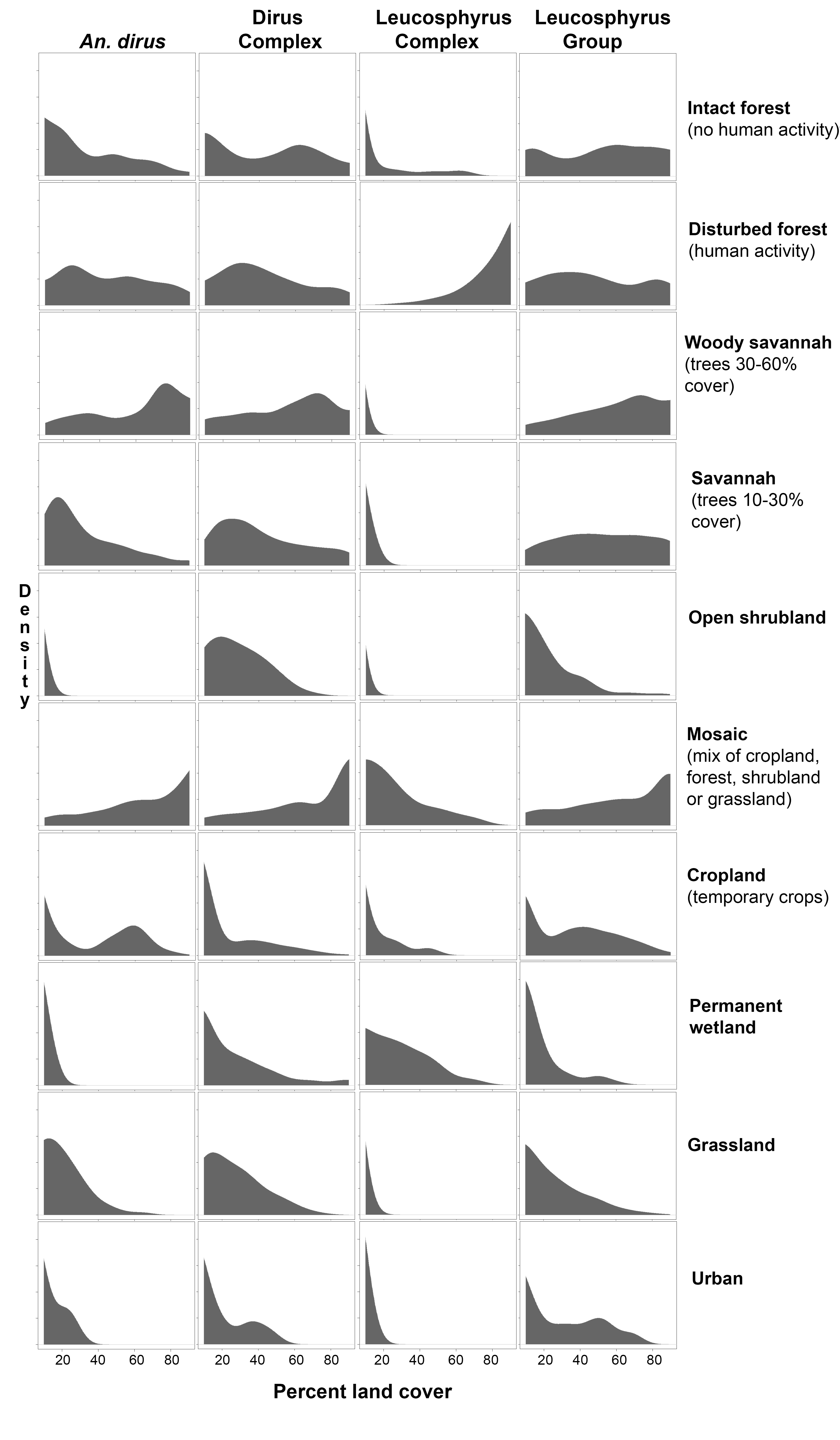

Supplement: Additional file 6: — Proportional land cover in areas with high predicted probability of species occurrence. Plots showing the relative density of pixels at each percentage land class coverage for all pixels where the probability of species occurrence was greater than 0.75, for each species. (DOCX 795 kb) [file 13071_2016_1527_MOESM6_ESM.docx]
